# A *Phytophthora sojae* CRN effector mediates phosphorylation and degradation of plant aquaporin proteins to suppress host immune signaling

**DOI:** 10.1371/journal.ppat.1009388

**Published:** 2021-03-12

**Authors:** Gan Ai, Qingyue Xia, Tianqiao Song, Tianli Li, Hai Zhu, Hao Peng, Jin Liu, Xiaowei Fu, Ming Zhang, Maofeng Jing, Ai Xia, Daolong Dou

**Affiliations:** 1 Key Laboratory of Plant Immunity, Academy for Advanced Interdisciplinary Studies, College of Plant Protection, Nanjing Agricultural University, Nanjing, China; 2 Institute of plant protection, Jiangsu Academy of Agricultural Sciences, Nanjing, China; 3 Department of Crop and Soil Sciences, Washington State University, Pullman, United States of America; University of Dundee, UNITED KINGDOM

## Abstract

*Phytophthora* genomes encode a myriad of Crinkler (CRN) effectors, some of which contain putative kinase domains. Little is known about the host targets of these kinase-domain-containing CRNs and their infection-promoting mechanisms. Here, we report the host target and functional mechanism of a conserved kinase CRN effector named CRN78 in a notorious oomycete pathogen, *Phytophthora sojae*. CRN78 promotes *Phytophthora capsici* infection in *Nicotiana benthamiana* and enhances *P*. *sojae* virulence on the host plant *Glycine max* by inhibiting plant H_2_O_2_ accumulation and immunity-related gene expression. Further investigation reveals that CRN78 interacts with PIP2-family aquaporin proteins including NbPIP2;2 from *N*. *benthamiana* and GmPIP2-13 from soybean on the plant plasma membrane, and membrane localization is necessary for virulence of CRN78. Next, CRN78 promotes phosphorylation of NbPIP2;2 or GmPIP2-13 using its kinase domain *in vivo*, leading to their subsequent protein degradation in a 26S-dependent pathway. Our data also demonstrates that NbPIP2;2 acts as a H_2_O_2_ transporter to positively regulate plant immunity and reactive oxygen species (ROS) accumulation. Phylogenetic analysis suggests that the phosphorylation sites of PIP2 proteins and the kinase domains of CRN78 homologs are highly conserved among higher plants and oomycete pathogens, respectively. Therefore, this study elucidates a conserved and novel pathway used by effector proteins to inhibit host cellular defenses by targeting and hijacking phosphorylation of plant aquaporin proteins.

## Author summary

CRN effectors are conserved in diverse pathogens of plants, animals, and insects, and highly expanded in *Phytophthora* species. Nevertheless, little is known about their functions, targets, and action mechanisms. Here, we characterized a kinase-domain-containing CRN effector (CRN78) in a notorious oomycete pathogen, *P*. *sojae*. CRN78 is a virulence-essential effector of *P*. *sojae* infection, and acts via suppression of plant H_2_O_2_ accumulation and defense gene expressions. We demonstrated that CRN78 might interact with plant PIP2-family aquaporin proteins, including *N*. *benthamiana* NbPIP2;2 and soybean GmPIP2-13, and regulate their phosphorylation, resulting in subsequent 26S-dependent protein degradation. Furthermore, we revealed that NbPIP2;2 was an apoplast-to-cytoplast H_2_O_2_ transporter and positively regulated plant immunity and ROS accumulation. Importantly, this phosphorylation may be highly conserved in many plant aquaporin proteins. Thus, this study identifies a virulence-related effector from *P*. *sojae* and a novel plant immunity-related gene, and reveals a detailed mechanism of effector-mediated phosphorylation and degradation of plant aquaporin proteins.

## Introduction

Unlike mammals, which can utilize specialized immune cells circulating in the bodies to combat pathogens, plants mainly rely on the innate immunity response in individual cells and sophisticated signal transduction networks to systematically counteract pathogen invasions [1]. When challenged by pathogens, protein receptors localized on the plant cell membrane can detect the conserved pathogen-associated molecular patterns (PAMPs) of pathogen molecules, and subsequently activate pattern-triggered immunity (PTI) [2]. The most commonly activated immune pathways include the generation of reactive oxygen species (ROS), calcium influx, mitogen-activated protein kinase (MAPK) cascade, and the production of defense-related hormones [3]. For successful colonization in host, virulent pathogens deliver diverse groups of effectors into plant cells to subvert host immunity [4,5]. Oomycete plant pathogens mainly secrete the following two classes of effectors: apoplastic effectors including enzyme inhibitors and small cysteine-rich proteins, and cytoplasmic effectors, such as RXLRs and CRNs (crinkling and necrosis proteins) [5].

CRNs, which are named after a distinctive leaf-crinkling phenotype observed upon ectopic expression, constitute a major family of effector proteins in oomycetes and have been recently reported as a protein family which widely exists in the eukaryotic taxon [6]. They usually contain a highly variant C-terminal domain, and two conserved motifs, LXLFLAK and HVLVVVP, in its N terminal [7,8]. Within the large reservoir of CRN effectors found in oomycetes and fungi, the studies on virulence mechanisms are only limited to several CRNs. A *Phytophthora sojae* effector, CRN108, inhibits the expression of plant heat shock proteins by targeting the host DNA directly to enhance host susceptibility [9]. CRN63 and CRN115 in *P*. *sojae* promote pathogenicity by interacting with host catalases to manipulate H_2_O_2_ homeostasis [10]. AeCRN13 of *Aphanomyces euteiches* and BdCRN13 of *Batrachochytrium dendrobatidis* promote host susceptibility by inducing nuclear DNA damages [11]. Thus far, most of the reported CRNs function in the nucleus of plant host cells [7,12], and the nuclear localizations are essential for the effector activity of CRNs. For example, the *P*. *infestans* effector CRN8 can be transported into the host nucleus by the nuclear pore complex importin-α [7] and cause cell death therein [13]. CRN108 and CRN63 from *P*. *sojae* also exert their functions in the plant cell nucleus [9,10]. Currently, it remains unclear whether CRNs can perform effector roles within different subcellular localizations, apart from the nucleus.

In addition to the conserved N-terminal motifs, LXLFLAK and HVLVVVP, many CRN effectors are predicted to harbor kinase domains [8,12,14,15]. For instance, the kinase-domain-containing CRN8 from *P*. *infestans* suppresses host defense and induces cell death [13]. However, molecular functions and mechanisms of action of these CRNs remain underexplored. Phosphorylation of host proteins by using the kinase-domain-containing effectors is a common strategy for plant bacteria pathogens to promote infection. The bacterial pathogen *Xanthomonas euvesicatoria* secretes a type III effector HopAU to phosphorylate plant MAP kinase signaling component MKK2 [16]. Bacterial effector HopBF1 phosphorylates the host chaperone HSP90 to inhibit its ATPase activity [17]. In oomycetes, the RXLR effector PexRD2 from *P*. *infestans* inhibits host resistance response by perturbing the phosphorylation of MAPKs [18]. However, it is unknown whether oomycete effectors also phosphorylate host targets or hijack this process to promote infection.

Plasma membrane intrinsic proteins (PIPs) belong to a subclass of membrane-intrinsic aquaporins proteins (AQP) that regulate the movement of water and small uncharged molecules [19–21]. The physical roles of aquaporins in plant development, growth, and stress responses have been extensively studied, but researches on the plant-pathogen interactions are rather scarce [22]. Recent literatures have revealed that plant PIPs can transport H_2_O_2_ in heterologous systems, and can be used by plant bacterial pathogens to facilitate infections [23–25]. In rice, Hpa1 produced by *Xanthomonas oryzae* interacts with plant OsPIP1;3 to promote the translocation of effectors [26]. Another PIP1 aquaporin protein of *Arabidopsis thaliana*, AtPIP1;4, acts as a H_2_O_2_ transporter upon pathogen infection to mediate H_2_O_2_ from apoplast to cytoplasm [27]. PIP proteins are also components of stomatal complexes, and they modulate the stomatal aperture [28,29]. For example, AtPIP2;1 facilitates stomatal closure triggered by ABA and pathogen invasion by promoting H_2_O_2_ generation in guard cells [30]. Though there are only a few reports on the involvement of aquaporin proteins in plant-pathogen interaction, these reports provide a glimpse into the importance of these proteins.

In this study, we identified a CRN effector, CRN78, from an important soybean pathogen, *P*. *sojae*, and demonstrated that it could inhibit plant immunity and ROS accumulation during infection. It associates with plant aquaporin proteins on the plasma membrane, and promotes their phosphorylation in a kinase dependent manner. This leads to a 26S-dependent degradation of the aquaporin proteins and subsequent reduction of ROS accumulation, thus facilitating the pathogen infection. This research proposed a novel virulent mechanism used by *P*. *sojae* via producing a CRN effector that manipulates phosphorylation of plant aquaporin proteins to inhibit ROS pathway, which may be adapted by other oomycete pathogens.

## Results

### *P*. *sojae* CRN78 promotes oomycete pathogen infection and inhibits plant immunity

*Phytophthora* genomes encode multiple kinase-domain-containing CRN effectors, but the functions of these CRNs on pathogen infection remain unclear [8,12,14,15]. In this study, a total of seven CRN effectors with kinase domains were identified in the genome of *P*. *sojae*, and one effector named CRN78 which contains a secretion signal peptide (SP), typical LXLFLAK and HVLVVVP motifs at its N terminal, and a predicted serine/threonine kinase domain, was selected for further analysis (S1A Fig). We first performed a yeast invertase secretion assay to functionally validate the predicted SP in CRN78 using the Avr1b SP and the pSUC2 empty vector as positive and negative controls, respectively [31]. Both SPs from CRN78 and Avr1b enabled yeast growth on the YPRAA media and exhibited red color with 2, 3, 5-triphenyltetrazolium chloride (S1B Fig), thereby confirming the secretory function of CRN78 SP. Collectively, these results indicated that CRN78 was a secreted effector.

**Fig 1:**
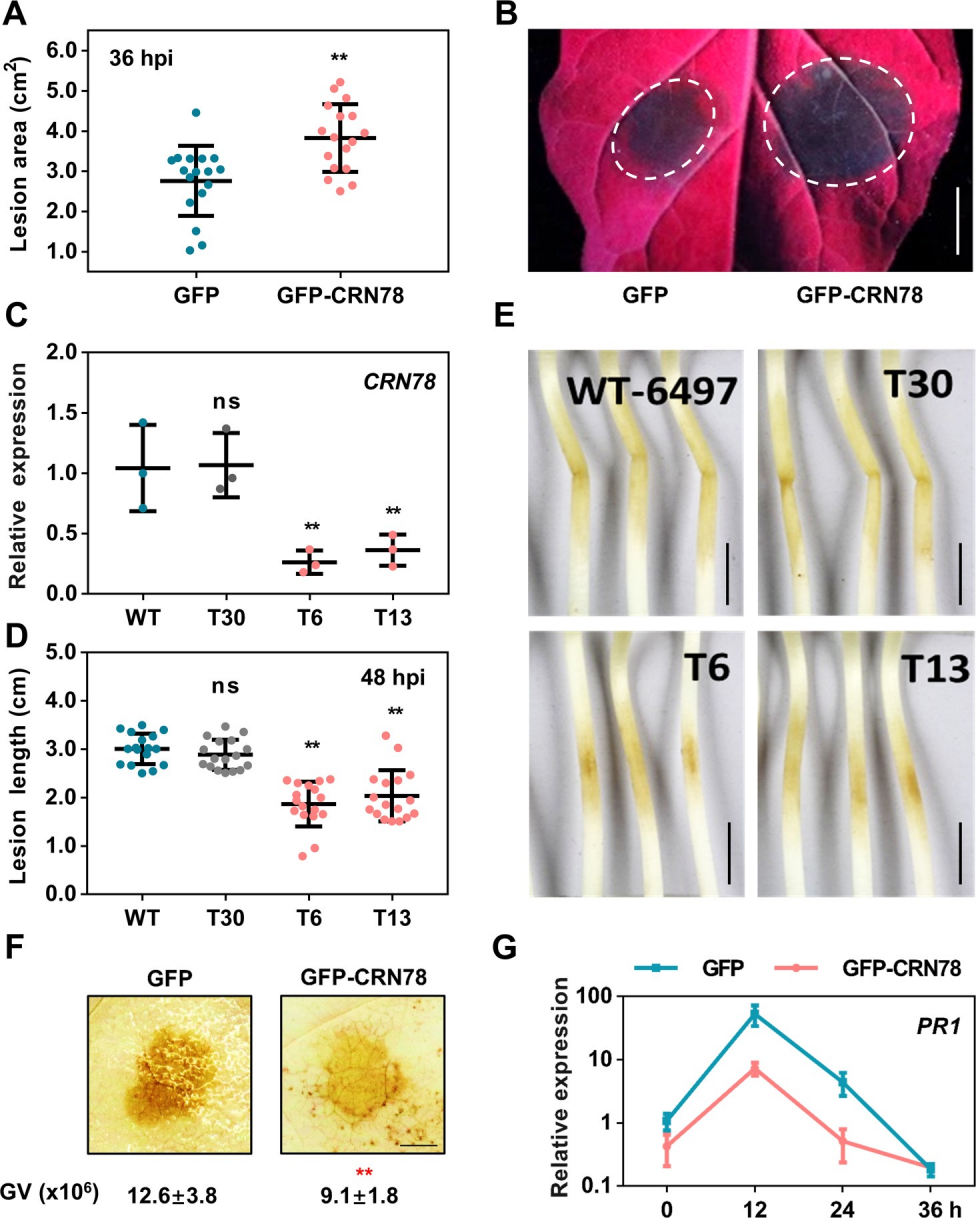
*P*. *sojae* CRN78 promotes oomycete pathogen infection and inhibits plant immunity. **(A and B) Enhanced *P*. *capsici* infection in *N*. *benthamiana* leaves expressing *CRN78*.** The infiltrated area expressing *GFP* or *GFP-CRN78* was infected with *P*. *capsici*. Lesion areas at 36 hpi (**A**) were calculated from three independent biological replicates using at least five leaves in each replicate (mean ± SD; n = 17; **, *P* < 0.01, Student’s *t*-test). The typical photographs (**B**) were taken at 36 hpi under UV light. **(C) Relative transcript levels of *CRN78* in different transgenic *P*. *sojae* lines.** WT: wild type P6497; T6 and T13: *CRN78*-silenced transformants; T30; a non-silenced transformant with same construct. The transcript levels were analyzed by qRT-PCR and normalized to that in the WT using the *actin* gene as an internal reference (mean ± SD; n = 3; **, *P* < 0.01 compared with the WT; Student’s *t*-test). **(D and E) Reduced lengths of lesions on etiolated soybean hypocotyls inoculated with the *CRN78*-silenced *P*. *sojae*.** Soybean cv. Williams was inoculated with ~100 zoospores of the *P*. *sojae* lines indicated. Lesion lengths (**D**) were analyzed at 48 hpi (mean ± SD; n > 15, **, *P* < 0.01 compared with WT; Student’s *t*-test). The typical phenotypes of lesions have been shown. Bar: 1 cm. **(F) ROS accumulation in *N*. *benthamiana*.** *P*. *capsici* was inoculated at 36 h post infiltration. DAB staining was conducted at 12 hpi. Integral grey value of DAB stained region was shown below (mean ± SD; n = 6, *P* < 0.01; Student’s *t*-test). **(G) Relative transcript levels of *NbPR1*.** The transcript levels of *NbPR1* in *N*. *benthamiana* leaves expressing *GFP* or *CRN78* were analyzed by qRT-PCR with *actin* as the internal reference (mean ± SD; n = 3).

To analyze the virulence of CRN78, *N*. *benthamiana* leaves overexpressing *GFP-CRN78/GFP* were challenged with *P*. *capsici*. The data showed that leaves with *GFP-CRN78* developed significantly larger lesion areas when compared to leaves with *GFP* control (Fig 1A and 1B). Western blot analysis demonstrated the expression of *GFP-CRN78* in *N*. *benthamiana* (S1C Fig). Additionally, stable transgenic *Arabidopsis* plants overexpressing *GFP-CRN78* (S1D Fig) were also produced and infected with *P*. *capsici* zoospores. The *GFP-CRN78-*transgenic leaves exhibited significantly larger lesion size in comparison with *GFP*-transgenic lines (S1E and S1F Fig). Together, these results revealed that *P*. *sojae* CRN78 decreased plant resistance to *P*. *capsici*.

Next, we investigated the role of CRN78 in the interaction between *P*. *sojae* and its native host soybean. In the first 48 h of *P*. *sojae* infection, the *CRN78* gene was significantly and highly expressed at both early and late stages of 4–8 and 24–36 hpi (S1G Fig). We then generated two stable *CRN78*-silencing *P*. *sojae* mutants, T6 and T13, with approximately 70%-75% reductions of the *CRN78* expression, and one control line T30 in which no silencing of *CRN78* was observed after transformation of the same construct (Fig 1C). When using these three lines and the wild-type *P*. *sojae* (WT-6497) to challenge soybean, T6- and T13-infected hypocotyls exhibited significantly shorter lesion lengths than T30 and WT-6497 (Fig 1D and 1E). Taken together, these results suggested that CRN78 was required for the virulence of *P*. *sojae* in interaction with soybean.

Unlike previous studies where kinase effector CRN8 was reported to induce cell death in *N*. *benthamiana* [13], transient expression assay and ion leakage analysis indicated that CRN78 did not induce cell death in *N*. *benthamiana* (S2A and S2B Fig). To explore the molecular mechanisms of CRN78-mediated pathogenicity, we used DAB staining assay to investigate H_2_O_2_ accumulation, a common immune pathway against plant pathogens, and our data revealed that transient expression of CRN78 in the leaves of *N*. *benthamiana* inhibited H_2_O_2_ accumulation in response to *P*. *capsici* infection (Fig 1F). Further, the transcripts of three well-known plant immunity marker genes, *PR1* (*Pathogenesis-related protein 1*), *PR2*, and *PDF1*.*2* (*PLANT DEFENSIN 1*.*2*) [32], in *N*. *benthamiana* leaves expressing *CRN78* or *GFP*, were analyzed using qRT-PCR. We found that the presence of *CRN78* in leaves reduced the expression level of all the three genes at both 12 and 24 hpi (Figs 1G, S2C and S2D). Thus, these results suggested that CRN78 enhanced pathogen virulence by inhibiting plant ROS accumulation and defense-related gene expression.

### CRN78 physically interacts with the *N*. *benthamiana* AQP NbPIP2;2

**Fig 2:**
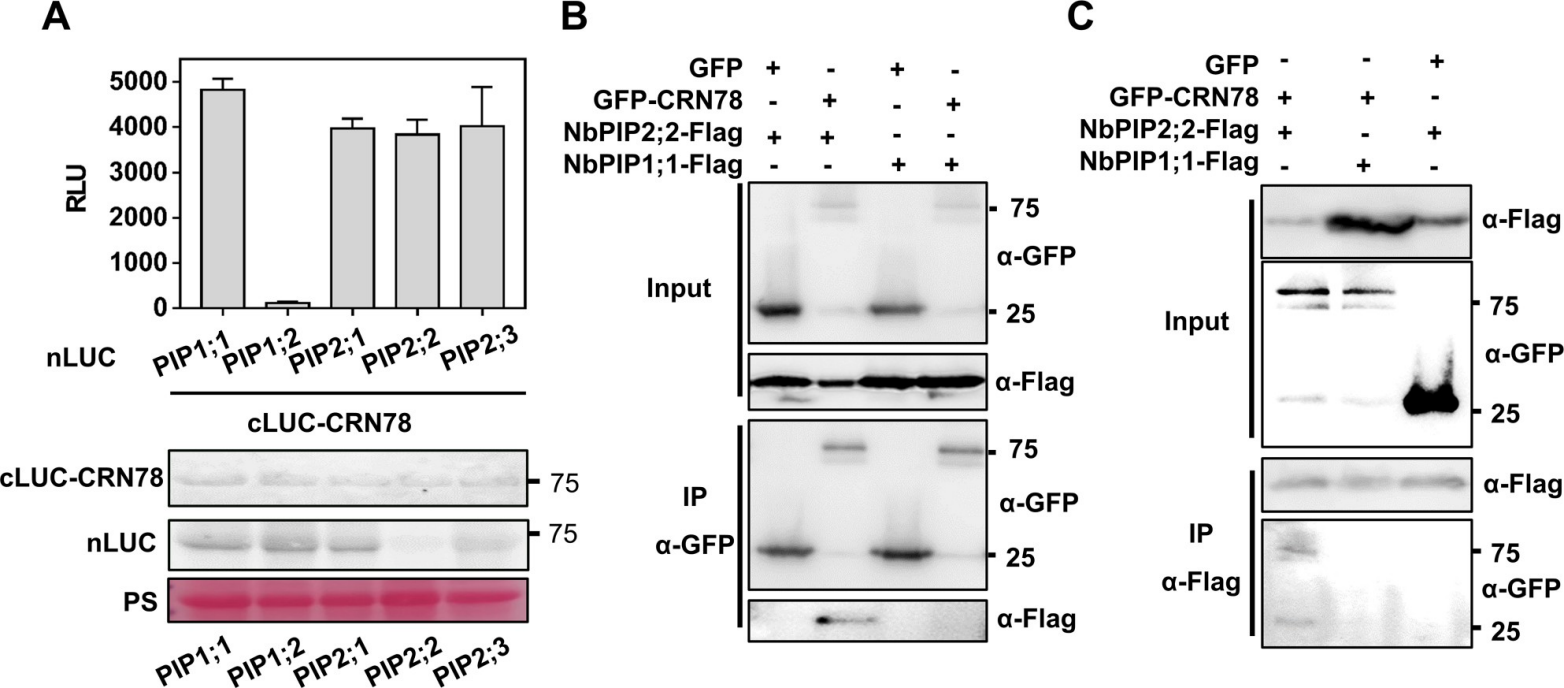
CRN78 physically interacts with the *N*. *benthamiana* AQP NbPIP2;2. **(A) CRN78 dynamically interacts with NbPIP2;2**. Luciferase complementation assay was performed on *N*. *benthamiana* plants by *Agrobacterium*-mediated transient expression of the indicated constructs. Relative luminescence units (RLU) of each combination are shown at the top (mean ± SD; n = 6). Protein expression is shown at the bottom. **(B and C) Verification of the interaction between CRN78 and NbPIP2;2 with the Co-IP assay.** Total proteins were extracted from *N*. *benthamiana* leaves expressing the indicated proteins. The immune complexes were immunoprecipitated with an α-FLAG or α-GFP beads, and the bound protein was detected by immunoblotting.

To identify the host targets of CRN78, *GFP-CRN78* was transiently expressed in *N*. *benthamiana* leaves followed by immunoprecipitation (IP) using anti-GFP affinity beads. GFP and an unrelated GFP-CRN108 protein were adopted as negative controls. Comparative mass spectrometry assay identified 158 putative proteins specifically associated with CRN78 (S3 Fig). Among these candidate targets of CRN78, AQPs represented the largest group, and therefore were selected for further analysis (S3 Fig). Five PIP AQPs were identified from the list and named as NbPIP1;1, NbPIP1;2, NbPIP2;1, NbPIP2;2, and NbPIP2;3 based on their phylogenetic relationships with the *Arabidopsis* counterparts (S4A Fig). Their respective interactions with CRN78 were further validated via the luciferase complementation assay. When fused with nLUC at the C terminal, all five PIPs, except NbPIP1;2, showed interactions with cLUC-CRN78 in *N*. *benthamiana* leaves (Fig 2A). No luminescence signal could be detected in the leaves expressing cLUC-CRN78 or PIPs-nLUC with negative controls (S5 Fig). Next, co-immunoprecipitation (Co-IP) assay was performed to confirm the interaction between NbPIP2;2 and CRN78. The results clearly showed that NbPIP2;2 was significantly enriched in the CRN78 precipitates instead of GFP control (Fig 2B) and CRN78 was significantly enriched in the NbPIP2;2 precipitates rather than NbPIP1;1 (Fig 2C). Therefore, all evidence demonstrated that AQP NbPIP2;2 was a potential target of CRN78 in *N*. *benthamiana*.

### The localization of CRN78 on plasma membrane is required for CRN78 virulence

**Fig 3:**
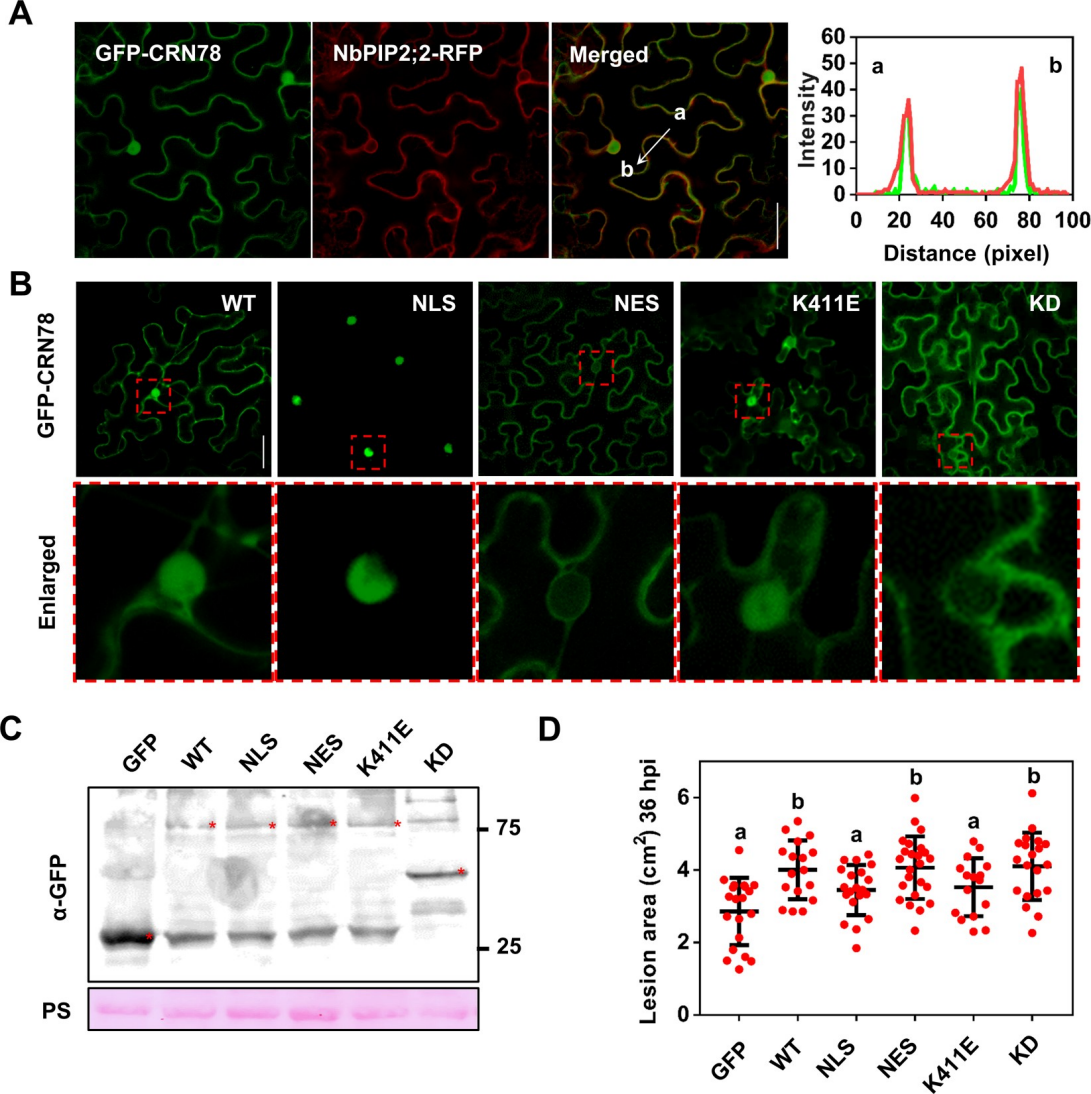
The localization of CRN78 on plasma membrane is required for CRN78 virulence. **(A) Co-localization of GFP-CRN78 and NbPIP2;2-RFP.** Confocal microscopy images were taken at 48 hours post infiltration. Scale bars = 50 μm. **(B) Subcellular localization of GFP-CRN78 and the mutants.** Confocal microscopy images were taken at 48 hours post infiltration. Detailed subcellular localization has been shown below. WT: GFP-CRN78^WT^; NLS: GFP-CRN78^NLS^; NES: GFP-CRN78^NES^; K411E: GFP-CRN78^K411E^; KD: GFP-CRN78^KD^. Scale bars = 50 μm. **(C) Immunoblot analysis.** The α-GFP antibody was used to detect expression of the indicated constructs. Equal loading of each sample is indicated by Ponceau staining of the Rubisco protein. **(D) Lesion areas of leaves expressing *GFP*, *CRN78* or the mutants upon *P*. *capsici* infection.** Lesion areas were calculated from three independent biological replicates using at least five leaves per replicate. Dots denote individual lesion area from each infection site (mean ± SD; n > = 16, Student’s *t*-test, *P* < 0.01).

To further confirm that NbPIP2;2 is the target protein of CRN78 on spatial locations, we investigated the subcellular localization of CRN78 *in planta* by transiently expressing *GFP-CRN78* in *N*. *benthamiana* leaves. GFP-CRN78 fluorescence signals were highly consistent with those of the nucleus and the plasma membrane markers (S6A and S6B Fig). Given that most published CRNs are located in the nucleus [7,12], we further validated the localization of CRN78 on the plasma membrane using a plasmolysis assay [33]. GFP-CRN78 was mainly detected in hechtian strands of plasmolyzed leaf tissues while GFP was not detected, indicating that GFP-CRN78 was localized in the plasma membrane (S6C Fig). Co-expression of GFP-CRN78 and NbPIP2;2-RFP indicated that NbPIP2;2 and CRN78 had same subcellular localizations in plasma membrane (Fig 3A). The same result was further confirmed by a bimolecular fluorescence complementation (BiFC) assay. The fluorescence signals indicated the co-localization between NbPIP2;2 and CRN78 in the cell periphery (S6D Fig). In contrast, no fluorescence signal was detected when NbPIP1;1-nYFP was co-expressed with cYFP-CRN78 (S6D Fig). To confirm that CRN78 interacted with NbPIP2;2 at the host plasma membrane, we conducted co-localization analysis of cYFP-CRN78, NbPIP2;2-nYFP, and membrane marker pm-RK. YFP fluorescence caused by interaction between cYFP-CRN78 and NbPIP2;2-nYFP was highly consistent with the pm-RK signal before or after plasmolysis treatment, suggesting that CRN78 co-localized with NbPIP2;2 at the host plasma membrane (S6E Fig). Thus, these findings demonstrate that the physical interaction between CRN78 and NbPIP2;2 mostly occurs in the plant plasma membrane.

To investigate whether the nucleus or the membrane localization contributed to the effector activity of CRN78, we generated two CRN78 mutants by adding a nuclear localization signal (GFP-CRN78^NLS^) or a nuclear export signal (GFP-CRN78^NES^) into the C-terminus of CRN78, respectively (S1A Fig). As shown in Fig 3B, GFP-CRN78^NLS^ was observed exclusively in the nucleus, while GFP-CRN78^NES^ was located outside the nucleus. With no remarkable difference of protein accumulation levels detected between CRN78 and its mutants (Fig 3C), GFP-CRN78^NES^ continued to produce significantly larger *P*. *capsici* lesions when compared with the GFP control, whereas GFP-CRN78^NLS^ lost its virulence (Fig 3D). Collectively, we suggest that the membrane localization of CRN78 is critical for its virulence instead of nucleus localization.

### NbPIP2;2 positively regulates H_2_O_2_ production and transportation

**Fig 4:**
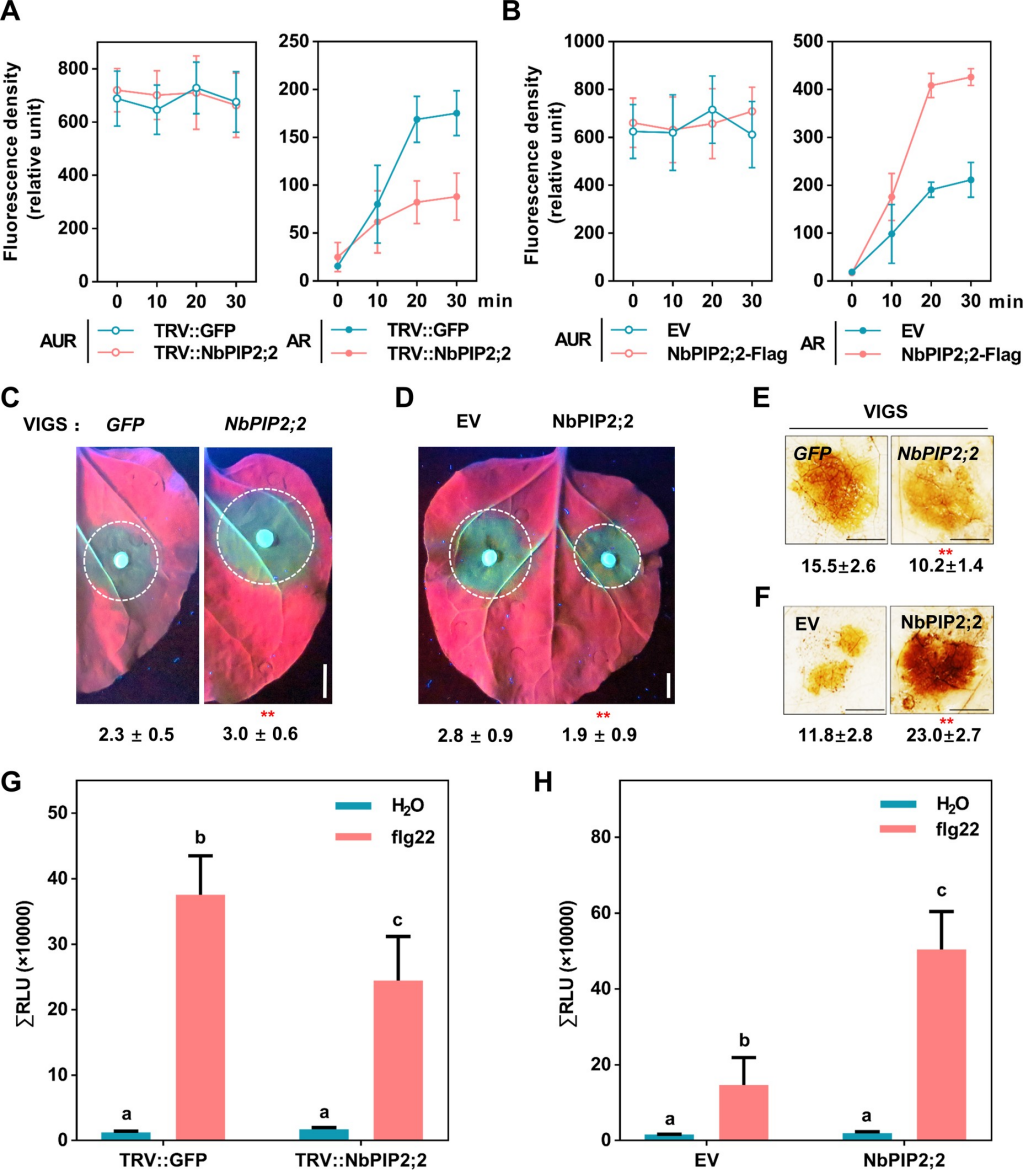
NbPIP2;2 acts as a H_2_O_2_ transporter to regulate plant ROS accumulation. **(A and B) Changes in the H**_**2**_**O**_**2**_**-probing fluorescence densities in the *NbPIP2;2*-silenced (A) or *NbPIP2;2*-overexpressing leaves (B).** The average AUR/AR florescence densities per 100 pixels of 20 randomly selected cells (relative unit) were quantified with the ImageJ in the indicated times. Error bar means SD. **(C) Enhanced *P*. *capsici* infection in the *NbPIP2;2*-silenced plants.** Photographs were taken at 36 hpi under UV light. Lesion areas shown below were calculated from three independent experiments with at least five leaves per replicate (mean ± SD; n = 18; **, *P* < 0.01, Student’s *t*-test). **(D) Reduced *P*. *capsici* infection in leaves expressing *NbPIP2;2*.** Lesion areas were calculated from three independent biological replicates with at least five leaves per replicate (mean ± SD; n > 15; **, *P* < 0.01, Student’s *t*-test). **(E and F) ROS accumulation in *NbPIP2;2*-silenced or *NbPIP2;2*-overexpressing leaves.** Leaves were collected for DAB staining at 12 hpi. Photographs were taken in white light. Integral grey value of DAB stained region was shown below (mean ± SD; n = 6, *P* < 0.01; Student’s *t*-test). **(G and H) ROS burst excited by flg22.** Total relative luminescence units (RLU) were detected over 30 min. Results shown are a representative of three independent experiments with eight replicates. Error bars indicate SD (mean ± SD; n > 10; *P* < 0.01, Student’s *t*-test).

To investigate the molecular role of NbPIP2;2 in plant immunity, the *NbPIP2;2* gene was silenced using PVX-based VIGS in *N*. *benthamiana* and confirmed with qRT-PCR assay (S7A and S7B Fig). Previous literatures reported that AQPs could transmit H_2_O_2_ [24,27]. In this study, we detected the ability of NbPIP2;2 to transport H_2_O_2_ by using Amplex Red (AR) and Amplex Ultra Red (AUR) probes, which can detect H_2_O_2_ in the cytoplast and the apoplast, respectively [27]. H_2_O_2_ was initially injected into leaves to establish consistency of the apoplast H_2_O_2_ levels, and AUR signals were comparable among leaves expressing different constructs (Figs 4A, 4B, S7C and S7D). In contrast, AR signals were much lower in the cells of *NbPIP2;2*-silenced plants (Figs 4A and S7C). Similarly, AR signals were enriched in cells expressing the NbPIP2;2 protein (Figs 4B and S7D). To exclude the possibility that the two dyes could induce the production of intracellular or intercellular H_2_O_2_, a mock treatment was done and the H_2_O_2_ level is unchangeable after the application of the dyes (S7E and S7F Fig). These results suggested that NbPIP2;2 regulated H_2_O_2_ transportation in *N*. *benthamiana*.

When infected with *P*. *capsici*, *NbPIP2;2*-silenced plants showed significantly larger disease lesions than *GFP*-silenced controls (Fig 4C). Consistent with this observation, transient expression of *NbPIP2;2* in *N*. *benthamiana* enhanced *P*. *capsici* resistance (Fig 4D). Therefore, these data demonstrated that during *P*. *capsici* infection, *NbPIP2;2* was a positive plant immunity regulator. We further evaluated the ROS burst using DAB staining method in *NbPIP2;2*-silenced leaves after *P*. *capsici* inoculation, and our result indicated that *NbPIP2;2*-silenced leaves exhibited much weaker ROS burst levels than *GFP* control (Fig 4E). Consistently, overexpression of *NbPIP2;2* resulted in dramatically higher ROS accumulation (Fig 4F).

The significantly increased ROS levels induced by NbPIP2;2 in DAB staining motivated us to explore whether that NbPIP2;2 also facilitated ROS production. To validate this hypothesis, we detected ROS production induced by the PAMP flg22 with or without NbPIP2;2, and found that ROS production was also positively correlated with *NbPIP2;2* expression levels (Fig 4G and 4H). Next, we investigated if NbPIP2;2-promoted H_2_O_2_ accumulation upon flg22 treatment using AR/AUR staining assay. Both AR and AUR probe signals were enriched in leaf cells containing NbPIP2;2 proteins (S8A and S8B Fig), and was impaired in *NbPIP2;2*-silenced leaves (S8C and S8D Fig), thereby indicating that NbPIP2;2 also promoted H_2_O_2_ accumulation in apoplastic space upon flg22. Taken together, we suggest that *NbPIP2;2* positively regulates both transportation and production of H_2_O_2_.

### CRN78 mediates phosphorylation of NbPIP2;2 at Ser276 and Ser279 *in planta*

As CRN78 contains a predicted serine/threonine kinase domain, it is reasonable to assume that CRN78 might mediate phosphorylation of NbPIP2;2. To test this hypothesis, we generated a dead-kinase mutant CRN78^K411E^ targeting the ATP-binding site of kinase domain, and a truncated (389^th^-574^th^) mutant CRN78^KD^, which only had kinase domain (S1A Fig). GFP-CRN78^K411E^ had similar localizations with the wild type protein, whereas GFP-CRN78^KD^ was only localized to the membrane (Fig 3B), suggesting that the nuclear localization of CRN78 was determined by its N terminal (Fig 3B). Despite the identical subcellular localization shared by GFP-CRN78 and GFP-CRN78^K411E^, GFP-CRN78^K411E^ lost the ability to promote *P*. *capsici* infection (Fig 3D). Meanwhile, GFP-CRN78^KD^ retained this infection-promoting ability, indicating that the full virulence of CRN78 solely depends on its kinase domain (Fig 3D).

**Fig 5:**
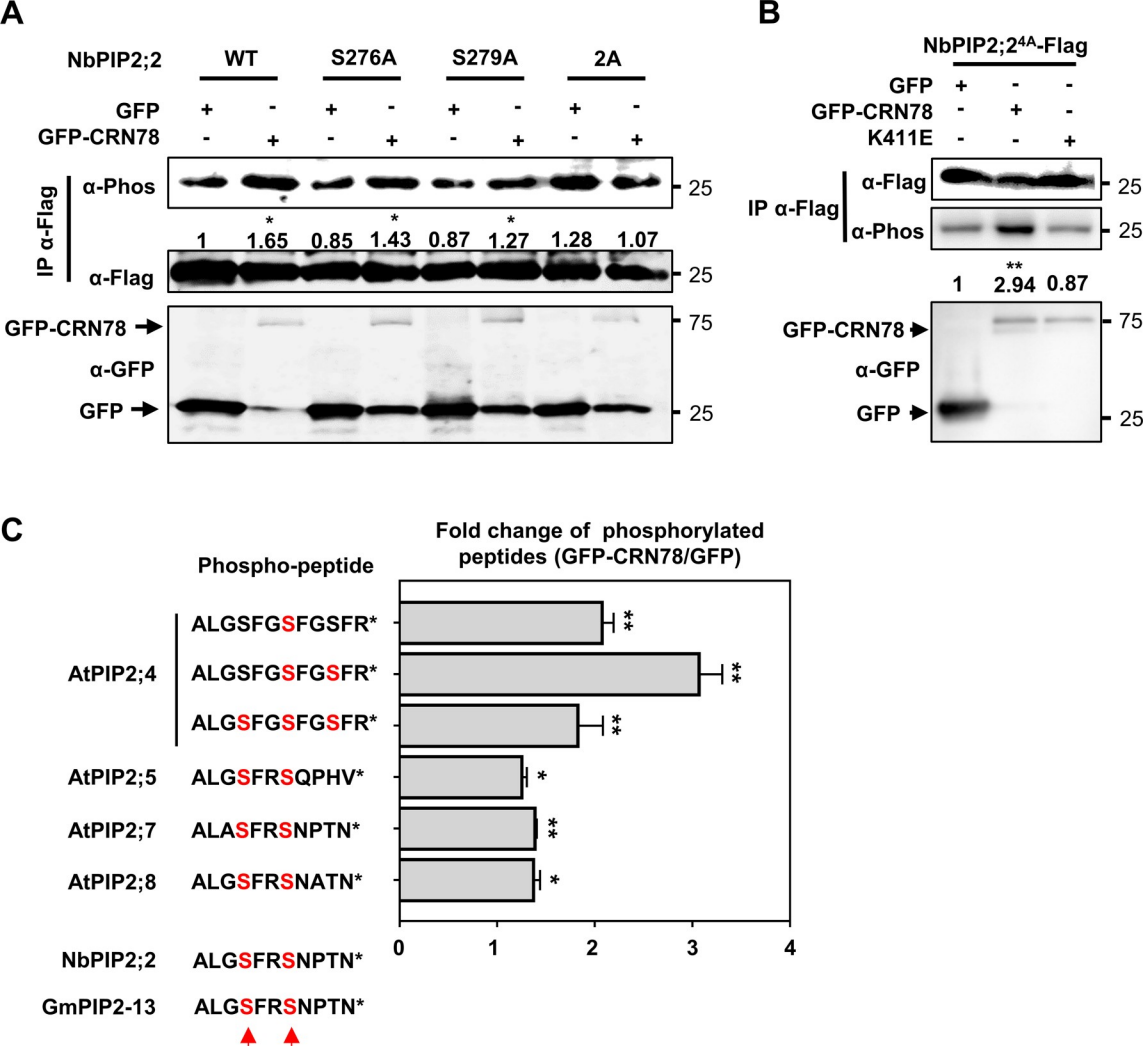
CRN78 phosphorylates NbPIP2;2 at Ser276 and Ser279 *in planta*. **(A) Phosphorylation of NbPIP2;2 by CRN78 *in vivo*.** *GFP-CRN78* or *GFP* together with *NbPIP2;2* or the indicated mutants were transiently co-expressed in *N*. *benthamiana* leaves, and α-Flag IP was performed to remove the background. Phosphorylation of NbPIP2;2 was conducted by α-Phos immunoblotting. Numbers below the blots represent the relative abundance of phosphorylated NbPIP2;2. α-Flag immunoblotting was used to show equal loading. **(B) Phosphorylation of NbPIP2;2 by CRN78 *in vivo*.** Numbers below the blots represent the relative abundance of phosphorylated NbPIP2;2. α-Flag immunoblotting was used to show equal loading. **(C) Enrichment of aquaporin phosphorylated peptides in samples with CRN78.** Six phosphorylated peptides of AtPIP2 proteins identified in the LC-MS/MS assay are shown (top left). Predicted phosphorylation sites in NbPIP2;2 and GmPIP2-13 are listed (bottom left). Bar graph (right) shows the relative fold changes of the six phosphorylated peptides (mean ± SD; n = 3; *, *P* < 0.05 **, *P* < 0.01, Student’s *t*-test).

We then co-expressed NbPIP2;2 and GFP-CRN78 in *N*. *benthamiana* leaves, and found that NbPIP2;2-Flag exerted a significantly stronger phosphorylation signal than that co-expressed with GFP (Fig 5A), indicating that NbPIP2;2 undergoes CRN78-mediated phosphorylation *in planta*. Native phosphorylation was detected in NbPIP2;2 without CRN78 (Fig 5A). To lower its native phosphorylation, we identified 4 putative phospho-residues in NbPIP2;2 and mutated them to alanine (NbPIP2;2^4A^) according to the known phospho-residues identified in AtPIP proteins (S9 and S10 Figs) [30,34]. The mutant showed extremely lower phosphorylation level, and CRN78 still greatly increased the phosphorylation level of NbPIP2;2^4A^ (Fig 5B). This result further supported that CRN78 mediated phosphorylation of NbPIP2;2 *in vivo*, and that there are other residues in NbPIP2;2 that got CRN78-mediated phosphorylation. To test whether the phosphorylation is dependent on the kinase activity of CRN78, we co-expressed NbPIP2;2^4A^ with CRN78^K411E^ and found CRN78^K411E^ could not elevate phosphorylation level of NbPIP2;2^4A^, indicating that phosphorylation of NbPIP2;2^4A^ by CRN78 is dependent on its kinase activity (Fig 5B). Collectively, we concluded that CRN78 mediated phosphorylation of NbPIP2;2 *in vivo*.

Next, we performed LC-MS/MS analysis with proteins extracted from stable *GFP-CRN78-* and *GFP*-transgenic *Arabidopsis* lines. Transgenic *Arabidopsis* was used to avoid the Agrobacterium interference induced by transient gene expression in *N*. *benthamiana*. After comparing normalized phospho-peptide counts between two sample groups, four AQPs (AtPIP2;4, 2;5, 2;7, and 2;8) were detected, and their peptides were significantly highly phosphorylated in the transgenic *Arabidopsis* expressing GFP-CRN78 (Fig 5C and S1 Data). All four AtPIP2s are highly homologous to NbPIP2;2 (Identity > 90%, *e* value < 1E-40) (S4A and S9 Fig) and the association between CRN78 and four AtPIP2 proteins *in planta* was confirmed with Luciferase complementation assays (S11A and S11B Fig). Therefore, all evidence revealed that four AtPIP2 proteins are all phosphorylation targets of CRN78 in *Arabidopsis*. We then used sequence alignment to search for the phosphorylation sites shared in NbPIP2;2 and AtPIP2 proteins, and the result detected two conserved amino acids, Ser276 and Ser279, in NbPIP2;2 which might be the two phosphorylation residues (Fig 5C). Hence, Ser276 and Ser279 were individually (NbPIP2;2^S276A^ and NbPIP2;2^S279A^) or simultaneously (NbPIP2;2^2A^) mutated to alanine (S10 Fig), and then were transiently co-expressed in *N*. *benthamiana* leaves with CRN78. Phospho-immunoblotting assay implied that compared to the GFP control, CRN78 still significantly increased the phosphorylation levels of both NbPIP2;2^S276A^ and NbPIP2;2^S279A^, but not that of NbPIP2;2^2A^ (Fig 5A). Thus, the results suggested that CRN78-mediated NbPIP2;2 phosphorylation occurs at both Ser276 and Ser279 *in planta*.

### CRN78 mediates phosphorylation of NbPIP2;2 at Ser279 and induces its degradation via a 26S-dependent pathway

**Fig 6:**
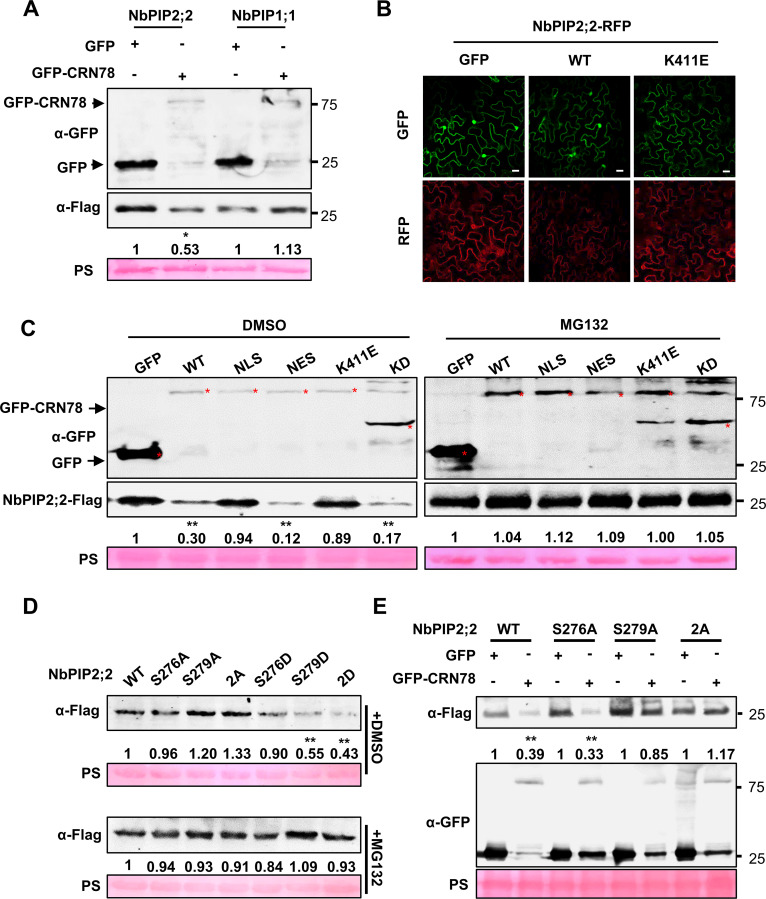
CRN78-mediated phosphorylation of NbPIP2;2 at Ser279 induces the degradation of NbPIP2;2. WT: GFP-CRN78^WT^; NLS: GFP-CRN78^NLS^; NES: GFP-CRN78^NES^; K411E: GFP-CRN78^K411E^; KD: GFP-CRN78^KD^. **(A) Degradation of NbPIP2;2 by CRN78 *in vivo*.** *GFP-CRN78* or *GFP* together with Flag-labeled *NbPIP2;2* or *NbPIP1;1* were transiently co-expressed in *N*. *benthamiana* leaves, and protein levels were analyzed by immunoblotting. Numbers below the blots represent the relative abundance of NbPIP2;2-Flag or NbPIP1;1-Flag. Ponceau staining was used to show equal loading. **(B) Live cell image assay.** Confocal microscopy images were taken at 48 hours post infiltration. **(C) Membrane localization and the kinase domain is important for CRN78 to reduce NbPIP2;2 abundance.** Protein levels under DMSO (left) or MG132 (right) treatment were analyzed by western blotting at 48 hours post infiltration. Ponceau staining was used to show equal loading. Numbers below the blots represent the relative abundance of NbPIP2;2-Flag. **(D) Degradation of NbPIP2;2 by phosphorylation at Ser279 *in vivo*.** Numbers below the blots represent the relative abundance of NbPIP2;2-Flag. Ponceau staining was used to show equal loading. Protein levels under DMSO (upper) or MG132 (below) treatment was shown by immunoblotting **(E) Protein accumulation levels of NbPIP2;2 mutants in the presence of CRN78.** Protein levels were analyzed by western blotting at 48 hours post infiltration. Ponceau staining was used to show equal loading. Numbers below the blots represent the relative abundance of NbPIP2;2-Flag.

Next, we aimed to illustrate how the interaction between two proteins affects the plant immunity. When we performed co-immunoprecipitation and co-localization assays with CRN78 and NbPIP2;2, it has come to our attention that the protein abundance of NbPIP2;2 was unusually low when it co-existed with CRN78 (Fig 2B). It inspired us to investigate whether CRN78 could reduce protein stability of NbPIP2;2. We compared the *in vivo* abundance of NbPIP2;2-Flag in agroinfiltrated *N*. *benthamiana* leaves when co-expressed with GFP-CRN78 and GFP, respectively. The non-interactor NbPIP1;1-Flag was selected as a negative control. Unlike GFP, the presence of GFP-CRN78 significantly reduced NbPIP2;2-Flag abundance (Fig 6A). In contrast, GFP-CRN78 did not affect NbPIP1;1-Flag levels (Fig 6A). CRN78-induced NbPIP2;2 degradation was further confirmed in a live-cell imaging assay using co-infiltrated *N*. *benthamiana* leaves. GFP-CRN78 significantly attenuated the red fluorescence of NbPIP2;2-RFP; however, it exhibited no fluorescence reduction effect on NbPIP1;1-RFP (Figs 6B and S12). Our data demonstrated that CRN78 promoted the degradation of NbPIP2;2 *in planta*. We then used the CRN78^K411E^ mutant that lost its kinase activity for further mechanism analysis. Results clearly revealed that the kinase activity of CRN78 is critical for the NbPIP2;2 degradation (Figs 6B and S12A), and the similar conclusion was drawn by Western blot assay data (S12B Fig). Overall, our evidence demonstrated that CRN78 promoted the degradation of NbPIP2;2 *in planta* through its kinase activity.

Referring to Fig 3D showing that CRN78 virulence is dependent on its membrane localization and the kinase domain, we assessed their association with the reduction of NbPIP2;2 abundance using CRN78 mutants. We found that the NbPIP2;2 reduction ability was retained in both GFP-CRN78^NES^ and CRN78^KD^, but not in GFP-CRN78^NLS^ or GFP-CRN78^K411E^ (Fig 6C), suggesting that both membrane localization and the kinase domain of CRN78 were required for the degradation of NbPIP2;2. To test whether the degradation of NbPIP2;2 was dependent on the 26S proteasome, we analyzed protein stability in the presence of MG132, a 26S proteasome inhibitor. The protein level of NbPIP2;2 could be restored upon MG132 treatment, indicating that CRN78 may promote the degradation of NbPIP2;2 in a 26S proteasome dependent pathway (Fig 6C). Afterward, we tested the potential association between NbPIP2;2 degradation and its CRN78-mediated phosphorylation at Ser276 and Ser279 with phospho-mimicking mutants created with Ser276 and Ser279 individually (NbPIP2;2^S276D^ and NbPIP2;2^S279D^) or simultaneously (NbPIP2;2^2D^) mutated to phosphorylation-mimicking aspartic acid (S10 Fig). In *N*. *benthamiana*, both NbPIP2;2^S279D^ and NbPIP2;2^2D^ exhibited significantly lower stability than that of the wild-type NbPIP2;2, whereas no apparent reduction was observed for NbPIP2;2^S276D^ accumulation (Fig 6D). The results suggested that NbPIP2;2 degradation was induced by phosphorylation at Ser279. Similarly, the low protein abundance of NbPIP2;2^S279D^ and NbPIP2;2^2D^ could be restored upon MG132 treatment (Fig 6D). When assessing the sensitivities of Ser276- and/or Ser279-mutated NbPIP2;2 proteins to CRN78-mediated degradation, both the wild-type NbPIP2;2 and NbPIP2;2^S276A^ were still degradable by CRN78 (Fig 6E), whereas neither NbPIP2;2^S279A^ nor NbPIP2;2^2A^ were subjected to CRN78-mediated degradation anymore (Fig 6E). Taken together, all experimental evidence demonstrated that phosphorylation of NbPIP2;2 at Ser279 was indispensable for its instability induced by CRN78 and that the subsequent degradation of NbPIP2;2 is dependent on 26S protease.

### Soybean GmPIP2-13 is subjected to CRN78-mediated phosphorylation and subsequent degradation

**Fig 7:**
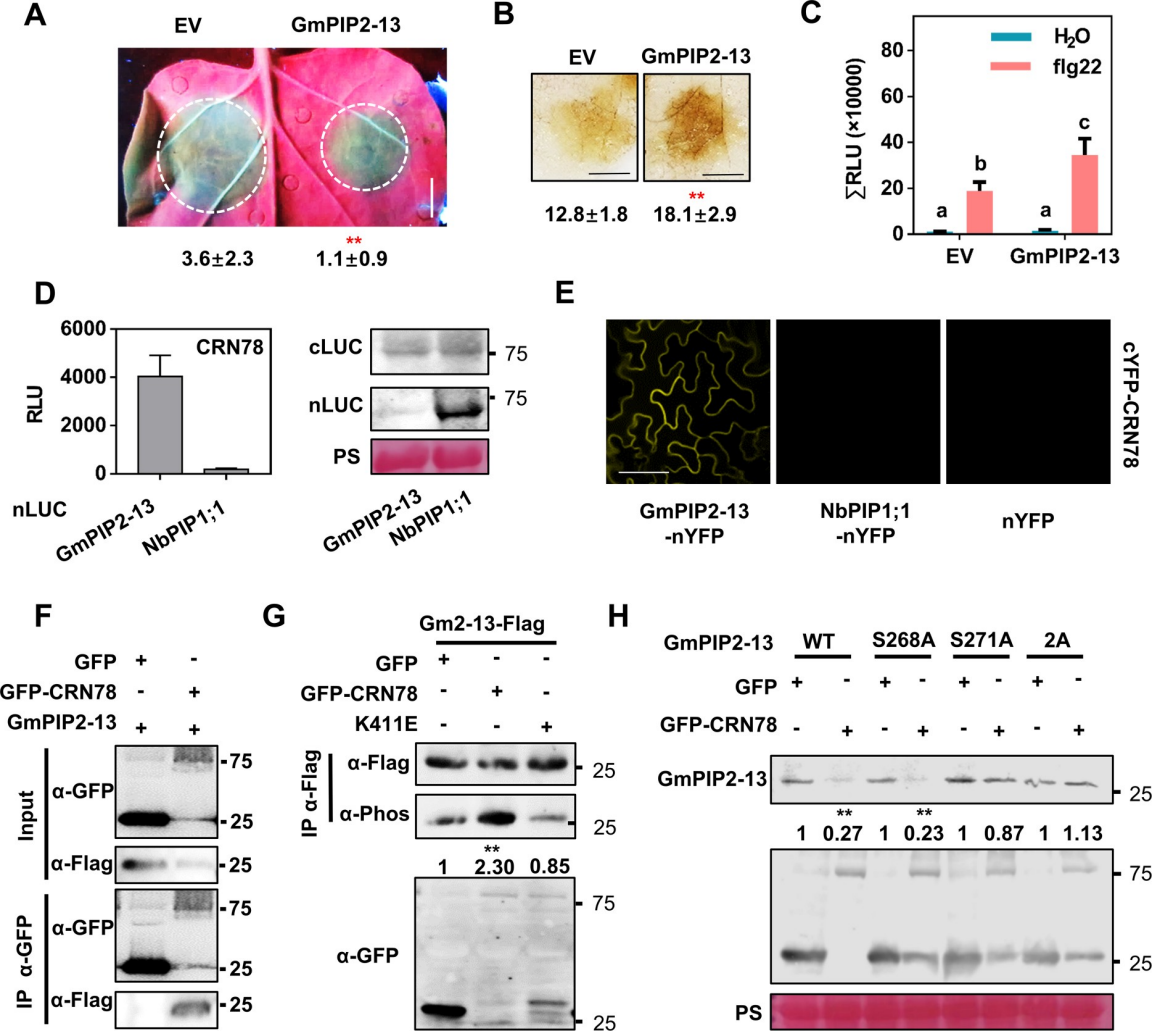
Soybean GmPIP2-13 is subject to CRN78-mediated phosphorylation and subsequent degradation. **(A) Reduced *P*. *capsici* infection in leaves expressing GmPIP2-13**. Photographs were taken at 36 hpi under UV light. Lesion areas were calculated from three independent biological replicates with at least five leaves per replicate (mean ± SD; n > 15; **, *P* < 0.01, Student’s *t*-test). **(B) Enhanced ROS accumulation in leaves expressing *GmPIP2-13*.** Leaves were collected for DAB staining at 12 hpi. Integral grey value of DAB stained region was shown below (mean ± SD; n = 6, *P* < 0.01; Student’s *t*-test). **(C) ROS burst elevated by flg22**. Total relative luminescence units (RLU) were detected at 30 min after treatment. The results shown are a representative of three independent experiments with eight replicates. Different letters indicate significant differences (mean ± SD; n > 10; *P* < 0.01, Student’s *t*-test). **(D) CRN78 interacts with GmPIP2-13.** Chemiluminescence signals were detected at 48 hours post infiltration. Proper protein expression is shown on the right. **(E) Interaction between CRN78 and GmPIP2-13 in a BiFC assay.** YFP fluorescence was observed 48 hours after infiltration. Scale bars: 20 μm. **(F) Interaction between CRN78 and GmPIP2-13 in a Co-IP assay.** Total proteins were extracted from *N*. *benthamiana* leaves expressing the indicated proteins. The immune complexes were immunoprecipitated with an α-GFP beads, and the bound protein was detected by immunoblotting. **(G) Phosphorylation of GmPIP2-13 by CRN78 *in vivo*.** Numbers below the blots represent the relative abundance of phosphorylated GmPIP2-13. α-Flag immunoblotting was used to show equal loading. **(H) Protein accumulation levels of GmPIP2-13 mutants in the presence of CRN78.** Protein levels were analyzed by western blotting at 48 hours post infiltration. Ponceau staining was used to show equal loading. Numbers below the blots represent the relative abundance of GmPIP2-13 -Flag.

Given that *N*. *benthamiana* is a model plant widely used in the laboratory but not the native host of *P*. *sojae*, we investigated the effector mechanism of CRN78 in soybean. We cloned *GmPIP2-13*, a homolog of *NbPIP2;2* in soybean (S4B Fig) and transiently expressed in the *N*. *benthamiana* leaves for conducting *P*. *capsici* infection assays. We found that similar to the findings obtained with NbPIP2;2, GmPIP2-13 significantly reduced leaf lesion (Fig 7A) and promoted ROS accumulation (Fig 7B) at the infection site of *P*. *capsici*. It also promoted flg22-triggered ROS burst (Fig 7C). These results demonstrated the positive role of GmPIP2-13 in plant immunity.

The luciferase complementation assay, BiFC assay and Co-IP assay confirmed that CRN78 interacted with GmPIP2-13 in the plasma membrane (Figs 7D, 7E and 7F). The observation that GmPIP2-13 phosphorylation was enhanced by CRN78 other than CRN78^K411E^ indicated the phosphorylation-mediated activity of CRN78 upon GmPIP2-13 (Fig 7G). We also mutated the potential phosphorylation residues (Ser268 and Ser271) to alanine based on the sequence alignment analysis (Fig 5C), and found that CRN78 only reduced the accumulation of GmPIP2-13^S268A^, but not GmPIP2-13^S271A^ or GmPIP2-13^2A^ (Fig 7H). These results indicate that CRN78 promotes GmPIP2-13 degradation by mediating its phosphorylation at Ser271, which is similar to the mechanism of NbPIP2;2 degradation triggered by Ser279 phosphorylation.

### CRN78 and PIP2;2 are conserved among oomycetes and higher plants, respectively

**Fig 8:**
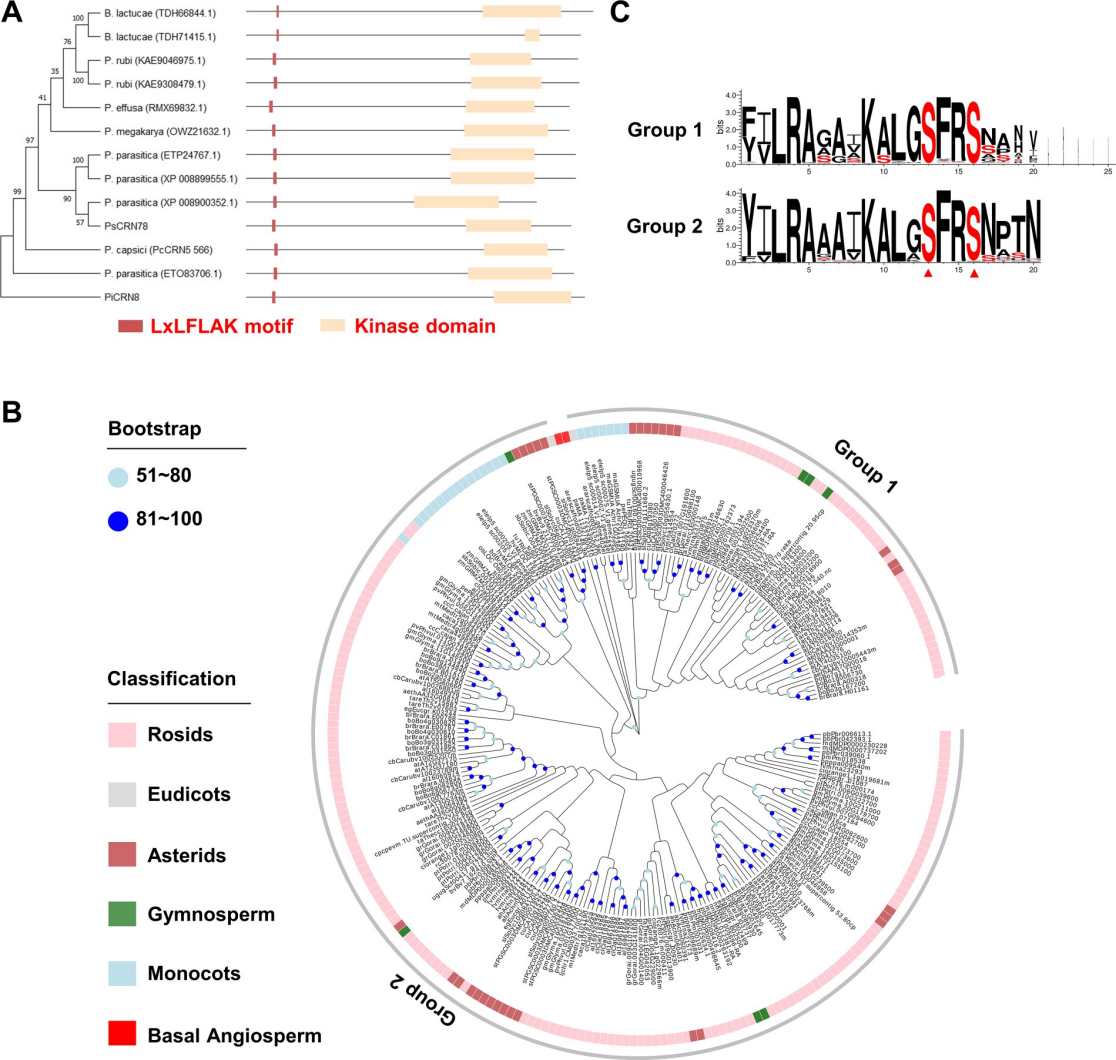
CRN78 and PIP2;2 are conserved among oomycetes and higher plants, respectively. **(A) The phylogenetic tree of CRN78-like proteins.** The phylogenetic tree is shown on the left. Diagram of conserved domains is shown on the right. **(B) Homologous proteins of PIP2;2**. The proteins were obtained by BlastP using NbPIP2;2 and GmPIP2-13 as queries (Identity > 50%, *E* value < 1E-5). Well-supported clades have been marked in light blue (bootstrap: 51 ~ 80) or blue (bootstrap: 81~100). **(C) Conserved motif flanking the phosphorylation site of PIP proteins.**

Using a genome database of 29 *P*. *sojae* strains as reported previously [35], we found that CRN78 is highly conserved among all 29 strains, indicating that CRN78 may be an essential effector for *P*. *sojae*. Sequence similarity searching showed that *CRN78* orthologous genes could be found in the genomes of other *Phytophthora* and downy mildew species (Figs 8A and S13). Importantly, the kinase domain is conserved in these CRN78-like proteins (Figs 8A and S13). Likewise, as the CRN78-acting target, NbPIP2;2 have homologs in 51 plant species from different major branches of the plant lineage (Fig 8B). The expansion of NbPIP2;2 homologs were also observed in the rosids and asterids with 6 homologs per species (S2 Data). In contrast, each monocot, gymnosperm or basal angiosperm species only have 2 homologs on average, and NbPIP2;2 homologs are completely absent in moss and green algae (S2 Data). These PIP2s can be divided into 2 groups based on their bootstrap values (> 50). NbPIP2;2 and GmPIP2-13 belong to group 2.

It was previously reported that conserved flanking sequences are critical for kinases to recognize the phosphorylation sites [36]. The CRN78-associated phosphorylation sites in PIP2s are also conserved in our analysis. Both the phospho-sites and their surrounding sequences in the C-terminal region are highly conserved throughout the whole clade, with a motif LGSFRS shared by almost all PIP2 homologs (Fig 8C and S3 Data). Overall, the phylogenetic analyses on both CRN78 and NbPIP2;2 homologs suggest that CRN78-induced PIP2 phosphorylation and subsequent degradation may be a common effector-target interaction approach adopted by oomycete pathogens to manipulate host plant immunity.

## Discussion

Amongst plant pathogens, members of the *Phytophthora* genus within oomycetes represent the most devastating pathogens, including *P*. *sojae*, *P*. *infestans*, *P*. *capsici*, and other species that affect almost every dicotyledonous crop plant [37,38]. These pathogens deploy two predominant classes of effectors, RXLRs and CRNs, to overcome host cellular defenses [5]. CRNs have been rarely studied, although they may play certain roles in triggering host susceptibility [5,6]. In this study, we functionally characterized CRN78, a novel protein kinase-domain-containing effector secreted by *P*. *sojae*. Essential for *P*. *sojae* virulence, CRN78’s activity depends on its plasma membrane localization and kinase domain. CRN78 physically interacts with the *N*. *benthamiana* aquaporin protein, NbPIP2;2, or soybean GmPIP2-13, each of which is an apoplast-to-cytoplast H_2_O_2_ transporter positively regulating plant immunity and ROS accumulation. CRN78 phosphorylates NbPIP2;2 *in planta* at both Ser276 and Ser279. The phosphorylation at Ser279 triggers protein instability of NbPIP2;2 in a 26S-dependent way. Phylogenetic analysis unveils that CRN78 and NbPIP2;2 are highly conserved in oomycetes and plants, respectively, suggesting that effector-mediated phosphorylation of plant aquaporin proteins might be an evolutionarily conserved mechanism.

CRNs were considered as a class of nuclear-localized effectors, and the nuclear localization was essential for the effector function in a number of cases [6,7,12]. For example, the *P*. *infestans* effector CRN8 can be transported into host nucleus by the nuclear pore complex importin-α to cause cell death [13]. CRN108 and CRN63 from *P*. *sojae* also exert their functions in plant cell nucleus [9,10]. Nevertheless, in this research, CRN78 executes its molecular function on the plant plasma membrane in violation of the defined nucleus localization feature (Figs 3A–3D and S6A–S6C). If the membrane location is mutated, CRN78 loses its virulence (Fig 3B, 3C and 3D). We also uncovered the virulence mechanism of CRN78 interacting with plant aquaporin proteins on the plant plasma membrane (Figs 3A, S6D and S6E). This finding unveils a novel acting pattern of CRN effectors and improves our knowledge on the molecular functions of CRN toward virulence.

There are several lines of evidence that plant bacterial pathogens secret kinase effectors to phosphorylate host proteins for pathogenicity [16,17]. For instance, bacterial effectors HopAU and HopBF1 phosphorylate a plant MAP kinase signaling component MKK2 and chaperone HSP90 [16,17]. However, to our knowledge, this phenomenon was not reported in oomycetes and fungi yet. In this study, we show that CRN78 harbors a conserved kinase domain and suppress plant immunity by mediating the phosphorylation of host PIP2s. As the AQPs phosphorylation enhanced by CRN78 is dependent on its kinase activity, we suspected that CRN78 phosphorylates AQPs directly. Furthermore, our assays in *N*. *benthamiana*, *Arabidopsis*, and soybean, together with the phylogeny results, suggest that this novel interaction mechanism between kinase-containing effectors and their host targets could be highly conserved.

Aquaporin proteins are widely distributed in all living organisms with important roles in physiological processes and stress responses [19–21,39]. In plants, AQPs such as AtPIP1;4 [27] and AtPIP2;1 [30] have been previously reported to transport H_2_O_2_ and thereby manipulate plant immunity. Here we confirmed that *N*. *benthamiana* NbPIP2;2, is also an apoplast-to-cytoplast H_2_O_2_ transporter (Figs 4A, 4B, S4C and S4D). When leaves were treated with exogenous H_2_O_2_, an increased accumulation of H_2_O_2_ could be detected inside cells (Fig 4A and 4B), indicating that H_2_O_2_ transportation was still occurring without pathogens. ROS are key regulators which participate in plant germination, growth, and flowering [40]. H_2_O_2_ transportation might be a mechanism by which PIP proteins regulate such biological processes. Interestingly, we also found that NbPIP2;2 can promote ROS accumulation (Figs 4G,4H and S8). It is still unknown how it positively regulates H_2_O_2_ production. It was believed that ROS signals from cytoplast could amplify apoplastic ROS [41]. Thus, we suspect that a proportion of H_2_O_2_ transportation by NbPIP2;2 from apoplast to cytoplast also acts as a signal to enhance ROS production.

How H_2_O_2_ transporter aquaporin proteins-induced immunity is overcome by plant pathogens for enhancing virulence is still an enigma. In this study, we revealed that *P*. *sojae* CRN78 was able to phosphorylate *N*. *benthamiana* PIP2;2 and soybean GmPIP2-13 at the C terminal to trigger their instability in a 26S-dependent pathway. This study provided the first-hand evidence that plant aquaporin proteins play important roles in the interaction between oomycete pathogens and hosts. Notably, in our phos-LC-MS/MS data, the phosphorylation of AtPIP2s at Ser279 could also be detected without CRN78 (Fig 5C). Further, our phos-WB data showed that the phosphorylation of NbPIP2;2 existed without CRN78 (Fig 5A and 5B), so we suspect that AQP phosphorylation and degradation may occur without pathogen infection. Furthermore, sequence flanking the phosphorylation site was conserved among higher plants (Fig 8B and 8C), implying that phosphorylation-dependent degradation of AQPs might be a common way used by plants to maintain homeostasis in AQPs. Continuous degradation of AQPs was also reported in AtPIP2;1, a homolog gene of NbPIP2;2 [42]. In overexpression transgenic plants, the ubiquitination of AtPIP2;1 could be detected without any treatment. As aquaporin proteins are important regulator of plant development, it is rational that their abundance is under precise management, and phosphorylation and ubiquitination may play a vital role in this process.

In mammals, phosphorylation is a well-studied process altering gating, trafficking, and abundance of aquaporin proteins [43]. Phosphorylation of human AQP2 at Ser261 leads to its endocytosis and degradation [44]. For example, Lysosomal Trafficking Regulator-Interacting Protein 5 (LIP5), a negative regulator of AQP2 protein stability, preferentially binds to the C-terminal of AQP2 when the Ser264 is phosphorylated [45,46]. In plant, phosphorylation of PIPs regulates their transport activity and trafficking [47–49], but less was shown about the link between phosphorylation and degradation. Here we showed that the phosphorylation at Ser279 mediated by CRN78 could induce degradation of NbPIP2;2. Ubiquitination and autophagy are involved in the degradation of PIPs [42,50]. Relocalization process was also shown closely related to abundance of PIPs in membrane [51,52]. Based on our data, the degradation of NbPIP2;2 proteins triggered by phosphorylation was dependent on 26S proteasome. Many important proteins were reported to interact with PIPs and regulated their trafficking and degradation [42,50–52]. For example, E3 ubiquitin ligase, Rma1H1, could interact with AtPIP2;1 and then promote its degradation. Further investigation on how C-terminal phosphorylation participates in the interaction between PIPs and these proteins is required.

In conclusion, *P*. *sojae* effector CRN78 interacts with plant PIP2 proteins to manipulate host cellular process using its kinase domain and membrane localization. These findings revealed a novel mechanism of CRN effector hijacking phosphorylation of host aquaporin proteins to overcome plant defense.

## Materials and methods

### Plasmid construct

CRN78 was amplified from *P*. *sojae* strain P6497. For the overexpression of CRN78 in *N*. *benthamiana* or *A*. *thaliana*, CRN78 or relative CRN78 mutants were cloned into pBinGFP2. *NbPIPs* and *GmPIP2-13* was amplified from *N*. *benthamiana* and *Glycine max* cv. Williams, respectively, and then inserted with C-terminal-fused Flag tag into pCambia1300. For BiFC assay, indicated genes were cloned into pCambia1300-cYFP or pCambia1300-nYFP. For yeast invertase secretion assay, the signal peptide regions of CRN78 and Avr1b were fused in-frame with the N-terminal of an invertase lacking the start codon and the signal peptide in the vector pSUC2. Plasma membrane marker pm-RK was reported previously [53]. For luciferase complementation assay, the coding sequences of indicated genes are cloned into pCAMBIA1300-35S-HA-Nluc-RBS or pCAMBIA1300-35S-Cluc-RBS. A start codon and a stop codon were added into the pCAMBIA1300-35S-HA-Nluc-RBS vector or pCAMBIA1300-35S-Cluc-RBS vector, respectively, to generate negative control vectors. To silence CRN78 in *P*. *sojae*, CRN78 was inserted into pTOR vector reversely. The anticipated construct is present and has not undergone any changes during cloning.

### Ion leakage assay and plasmolysis assay

Ion leakage induced by cell death was analyzed as per previously described methods [54]. In brief, three 9 mm-leaf disks were rinsed three times with distilled water and then soaked in 5 mL of distilled water for 3 h at room temperature. The conductivity of the solution was measured using a conductivity meter to generate value “A”. The leaf disks were then boiled for 25 min and the conductivity was measured again to generate value “B”. The electrolyte leakage was calculated as the percentage (%) of “A”/“B”. The assay was repeated for a total of three times.

In plasmolysis assay, 800 mM mannitol was injected into the leaves to induce plasmolysis. Photos were taken at 5 min after injection using a confocal laser-scanning microscope (Zeiss LSM700, Germany).

### Plant materials and transgenic *Arabidopsis* strains

*N*. *benthamiana* plants used in this study were grown in the greenhouse at a temperature of 25°C under a 16-h light/8-h dark photoperiod and 60% relative humidity for six weeks. Etiolated soybean seedlings (cv. Williams) were grown at 25°C in the dark for 4 days before harvesting for inoculation. *Arabidopsis* plants were grown at 23°C under a 12-h day/12-h night photoperiod and 60% relative humidity for 30 days.

To generate GFP-transgenic *Arabidopsis* plants, empty pBinGFP2 / pBinGFP2-CRN78 (without SP region) plasmid was transformed into *Agrobacterium* strain GV3101 and transferred to *Arabidopsis* wild-type (Col-0) plants using the standard *Agrobacterium*-mediated floral dip protocol [55]. The transgenic plants were screened on 1/2 MS medium containing 50 mg/L kanamycin to obtain positive transgenic plants. For each transgenic plant, two lines were obtained and T2 generation plants in which transgene expression was confirmed by Western blot were selected for experiments.

### Transient expression and VIGS in *N*. *benthamiana*

The indicated recombinant constructs were transformed into *Agrobacterium tumefaciens* strain GV3101. For infiltration, *Agrobacterium* strains were cultured at 28°C and 220 rpm for 48 h, and the cells were collected after centrifugation. The cells were washed and then re-suspended in 10 mM MgCl_2_ to an appropriate optical density (OD) at 600 nm (0.4–0.6). Five-week-old *N*. *benthamiana* leaves were infiltrated for transient expression. To inhibit 26S proteasome, 100 μM MG132 was injected into the leaves for 6 h.

For *Agrobacterium*-mediated VIGS, TRV vectors pTRV-RNA1 and pTRV-RNA2, namely pTRV-NbPIP2;2, pTRV-GFP (negative control), and pTRV-PDS (positive control), were introduced into *A*. *tumefaciens* strain GV3101 by electroporation. *Agrobacterium* suspensions containing pTRV-RNA1 and pTRV-RNA2 derivatives were mixed at an equal ratio and inoculated into the first pair of true leaves of 10-day-old soil-grown *N*. *benthamiana*. Treated *N*. *benthamiana* plants were maintained at 23°C under a 16-h light/8-h dark photoperiod for 2 weeks before being collected for assays.

### Plant pathogen strains and the inoculation assay

*P*. *sojae* transformation and the screening of putative transformants were performed using previously described methods [56,57]. *P*. *sojae* strain P6497 was grown in V8 solid media for 3 days and then transferred into pea broth medium. Mycelia were washed and placed in enzyme solution (0.4 M mannitol, 20 mM MES pH 5.7, 10 mM CaCl_2_, 20 mM KCl, 10 mg/mL β-1.3 glucanase, and 5 mg/mL cellulysin) and incubated for 40 min at 25°C and shaking at 100 rpm to obtain protoplasts. The protoplasts were then washed and resuspended in an equal volume of MMg solution (0. 4 M mannitol, 15 mM MgCl_2_, and 4 mM MES; pH 5.7). For 1 mL MMg solution, 25 mg transforming DNA was added. Then, three aliquots of 580 mL each of polyethylene glycol (PEG) solution (40% (v/v) PEG4000, 0.3 M mannitol, and 0.15 M CaCl_2_) were slowly added into the protoplast suspension and mixed gently. The protoplasts were incubated in pea broth medium overnight to regenerate and then grown on medium containing 25 μg/mL G418. The mycelia grown normally were transferred into medium containing 50 μg/mL G418 and selected for further confirmation. Silenced transgenic lines were screened by qRT-PCR. The *P*. *capsici* strain LT263, *P*. *sojae* strain P6497, and *CRN78*-silenced mutants were cultured and maintained at 25°C in the dark on 10% (v/v) V8 juice medium. For mycelium inoculation on *N*. *benthamiana*, 5-mm disks of 4-day growth medium were inoculated on leaves 24 h after infiltration. Inoculated leaves were photographed under UV light 36 or 48 hpi, and the lesion area was evaluated at the indicated time points. For zoospore inoculation on *Arabidopsis*, approximately 500 *P*. *capsici* zoospores were dropped at the center of each detached leaf, and then incubated in a growth room at 25°C in darkness. Inoculated leaves were photographed under UV light and lesion areas were evaluated at the indicated time points. To quantitatively test the virulence of *P*. *sojae* transformants, each etiolated soybean seedlings of cv. Williams were inoculated with approximately 100 *P*. *sojae* zoospores. Lesion lengths were measured after 36 h.

### Signal peptide secretion confirmation

The yeast signal sequence trap assay was performed as per previously reported methods [31]. The signal peptide regions of CRN78 and Avr1b were fused in-frame with the N-terminal of an invertase lacking the start codon and the signal peptide in the vector pSUC2, respectively. Recombinant constructs were transformed into yeast. Positive clones were selected in the CMD-W (minus Trp) media and then transferred to YPRAA plates. Successful growth of recombinant yeast cells in YPRAA plates indicated invertase secretion. Invertase secretion was also determined by monitoring the conversion of TTC to the insoluble, red-colored triphenylformazan when recombinant yeast cells were grown in the medium containing TTC.

### qRT-PCR analysis and bioinformatics analysis

For qRT-PCR analysis, total RNA was extracted by using an RNA-simple Total RNA Kit (Tiangen Biotech Co., Ltd., Beijing, China) according to the manufacturer’s instructions. *P*. *sojae* and *N*. *benthamiana* cDNA was synthesized with the HiScript II Q RT SuperMix for qPCR (Vazyme Biotech Co., Ltd., Nanjing, China). Real-time PCR was performed by using a SYBR Premix Ex Taq Kit (Takara Bio Inc., Shiga, Japan) on an ABI Prism 7500 Fast Real-Time PCR system following the manufacturer’s instructions. Expression levels were normalized to the expression of *NbACTIN* and *PsActin*, which are stably expressed reference genes in *N*. *benthamiana* and *P*. *sojae*, respectively. The primers used for qRT–PCR are listed in S4 Data. All the qRT-PCR results showed in this study were calculated from three independent biological replicates.

For bioinformatics analysis, sequences were aligned using the MUSCLE software [58]. Phylogenetic trees were constructed using the MEGAX software (https://www.megasoftware.net/) with Neighbor-Joining method (Bootstrap = 1000, *p-distance*, *pairwise deletion*) and visualized using EvolView v3 [59]. Weblogo were obtained using Weblogo 3 [60].

### Western blotting

To extract proteins from plant materials, leaves were frozen in liquid nitrogen and ground to a fine powder. For normal western blot assay, extraction buffer (50 mM HEPES, 150 mM KCL, 1 mM EDTA, and 0.1% Triton X-100; pH 7.5), supplemented with 1 mM DTT and protease inhibitor cocktail (Sigma-Aldrich, St. Louis, MO, USA), was used for protein extraction from plant materials. Anti-Flag (1:5, 000; #M20008; Abmart Inc., Shanghai, China), anti-GFP (1:5, 000; #M20004; Abmart), and anti-RFP (1:1, 000; #5f8; Chromo Tek GmbH, Munich, Germany) antibodies were used to bind the protein with indicated tag.

For phosphorylation detection, phosphatase inhibitors (Sigma-Aldrich) were used in extraction buffer. The total proteins of leaves which expressed indicated constructs were extracted. Then the NbPIP2;2 or GmPIP2-13 proteins were purified with anti-Flag affinity beads. The purified proteins were loaded equally in the gel, fractionated by SDS–PAGE and transferred into PVDF membrane. The membrane was then blocked using TBS (pH 7.4) containing 3% BSA for 1 h at 25°C and shaking at 40 rpm, followed by washing three times with TBST (TBS with 0.1% Tween 20). The phosphorylation levels of purified proteins were detected by using anti-phosphoserine antibodies (1:500; #ab9332; Abcam, Cambridge, MA, USA). Western blot bands were quantified with the ImageJ software (https://imagej.en.softonic.com/). The images were first converted to 8-bit format and smooth continuous background removed with 50 pixels as parameter in ImageJ. The whole image was then inverted, and the integral grey value of each band was calculated. The digital number shown below the western blot band was a ratio of integral grey value of such band to the control band.

### LC-MS/MS assay and identification of phosphorylated peptide

To identify putative targets of CRN78, *GFP-CRN78* was transiently expressed in the leaves of *N*. *benthamiana*. α-GFP IP was carried out as per previously described methods [61]. The immune precipitates were washed three times with the protein extraction buffer and then digested with trypsin. Tryptic peptides were analyzed by LC-MS/MS using Q-Exactive HF X (Thermo Fisher Scientific, Waltham, MA, USA).

To identify peptides phosphorylated by CRN78 in *Arabidopsis*, protein samples were extracted from GFP and GFP-CRN78 transgenic-plants (three replicates) using protein extraction buffer supplemented with phosphatase inhibitors. Protein samples were digested with trypsin followed by phosphopeptide enrichment and then analyzed by LC-MS/MS using Q-Exactive HF X (Thermo Fisher Scientific). Phosphorylated peptides of each sample were evaluated and were used in analysis.

### Luciferase complementation assay

The coding sequences of indicated genes were cloned into pCAMBIA1300-35S-HA-Nluc-RBS or pCAMBIA1300-35S-Cluc-RBS and were introduced into *A*. *tumefaciens* strain GV3101. *Agrobacterium* strains carrying the indicated constructs were infiltrated into *N*. *benthamiana* leaves. Leaf discs were taken 2 days later, incubated with 1 mM luciferin in a 96-well plate for 10 min, and luminescence was recorded with a microplate reader (BioTek, Beijing, China).

### BiFC assay and Co-IP assay

Different combinations of *Agrobacterium* GV3101 containing cYFP-CRN78, NbPIP1;1-nYFP, NbPIP2;2-nYFP, or GmPIP2-13-nYFP plasmids were injected into leaves of *N*. *benthamiana*; YFP signals were observed under a confocal laser-scanning microscope (Zeiss LSM700, Germany).

For Co-IP assay, indicated plasmid combinations were transiently expressed in *N*. *benthamiana* following the methods described above. α-GFP and α-Flag IPs were carried out as per previously described methods [55]. Total proteins were extracted at 48 hours post infiltration following the methods described above and then incubated with α-GFP (ChromoTek GmbH) or α-Flag beads (Abmart Inc.) overnight. The beads were collected by centrifuge and then washed five times with cold 1xTBS (containing 0.5% Triton X-100). Proteins were released from the beads by incubating at 100°C for 8 min with 50 μL 1xTBS. Immune precipitates were separated by SDS–PAGE gels and detected by immunoblotting with monoclonal α-GFP and α-Flag antibodies (Abmart Inc.).

### Oxidative burst assay, DAB staining, and apoplastic/cytoplastic H_2_O_2_ detection

For oxidative burst assay, soil-grown 5-week-old *N*. *benthamiana* leaves were sliced into 5-mm disks and incubated in 200 mL of water in a 96-well plate overnight. Then 1 mM flg22 in 200 mL of luminescence detection buffer (100 mM luminol and 20 mg/mL horseradish peroxidase) was added and the luminescence was recorded with a microplate reader (BioTek) for 30 min.

For DAB staining, 12 hours post inoculation of *P*. *capsici*, *N*. *benthamiana* leaves were collected and stained with 1 mg/mL DAB solution for 8 h in the dark. Then, the leaves were de-stained with 95% ethanol before light microscopy inspection. To quantify the results of DAB staining, the images were first converted to 8-bit format and then inverted. The integral grey value of each band was calculated by imageJ.

Apoplastic/cytoplastic H_2_O_2_ detection was performed on *N*. *benthamiana* leaves by staining with the ROS-probing dyes AR or AUR (Thermo Fisher Scientific) at a final concentration of 10 mM [27]. While AUR is impermeable to plasma membrane and only detects H_2_O_2_ in the apoplast, AR can penetrate plasma membrane and thus probe cytoplasmic H_2_O_2_. Solutions of dyes were injected 30 min earlier to the leaves to ensure sufficient diffusion into living cells. Pretreated leaves were then injected by 0.1 mM H_2_O_2_ or sprayed with 10 μM flg22, and were observed at 10-min intervals for 30 min with the Zeiss LSM700 laser scanning confocal microscope. The fluorescence emission of oxidized AR or AUR was excited using 543-nm argon laser and observed between 585 nm and 610 nm. The average AUR/AR florescence densities per 100 pixels of 20 randomly selected cells (relative unit) were quantified with the ImageJ software (https://imagej.en.softonic.com/). Quantification of the AR and AUR probing signals was restricted to cytoplasmic and apoplastic spaces, respectively.

## Supporting information

S1 Fig Characterization of CRN78. **(A)** Diagram of motifs in CRN78 and CRN78 mutants constructed in this study. **(B)** Yeast invertase secretion assay of the predicted CRN78 signal peptide. **(C)** Immunoblot analysis. The α-GFP antibody was used to detect expression of the indicated constructs. Equal loading of each sample is indicated by Ponceau staining of the Rubisco protein (PS). **(D-F)** Enhanced *P*. *capsici* infection in leaves expressing *CRN78*. 500 *P*. *capsici* zoospores were inoculated onto the leaves expressing GFP or GFP-CRN78. Photographs **(E)** were taken at 36 hpi under UV light. Lesion areas **(F)** were calculated from three independent biological replicates with at least five leaves per replicate (mean ± SD; n > 16; **, *P* < 0.01, Student’s *t*-test). **(G)** Relative transcript levels of *CRN78* during infection. Hypocotyls of etiolated soybean cv. Williams were challenged with zoospores of *P*. *sojae*, and RNA was extracted at indicated times. The transcript levels of CRN78 in *P*. *sojae* were normalized to the levels of *PsACTIN* gene. (mean ± SD; n = 3, *, *P* < 0.05, **, *P* < 0.01 compared with the sample of 0 hpi; Student’s *t*-test).
(TIF)

S2 Fig CRN78-induced phenotype in leaves of *N*. *benthamiana*. **(A)** CRN78 did not induce cell death in leaves of *N*. *benthamiana*. **(B)** Ion leakage assay in leaves expressing CRN78. **(C and D)** Relative transcript levels of *NbPR2* and *NbPDF1*.*2*. The transcript levels of *NbPR2* and *NbPDF1*.*2* in *N*. *benthamiana* leaves expressing *GFP* or *CRN78* were analyzed by qRT-PCR with *actin* as the internal reference (mean ± SD; n = 3).
(TIF)

S3 Fig The analysis of mass spectrometry data. Venn diagram of putative interactor of GFP/GFP-CRN78/GFP-CRN108 is shown on the left. The Protein frequencies of top 10 super families are shown on the right.
(TIF)

S4 Fig The phylogenetic tree of aquaporin proteins. **(A)** The phylogenetic tree of five NbPIPs identified in mass spectrum data with all aquaporins reported in *Arabidopsis*. The five NbPIPs is indicated in red. **(B)** The phylogenetic tree of five NbPIPs with all PIP2 proteins from soybean. The five NbPIPs are indicated in red and GmPIP2-13 is indicated in blue.
(TIF)

S5 Fig Negative controls in luciferase complementation assay. Luciferase complementation assay was performed on *N*. *benthamiana* plants by *Agrobacterium*-mediated transient expression of the indicated constructs. Relative luminescence units (RLU) of each combination are shown at the top (mean ± SD; n = 3). Proper protein expression is shown at the bottom.
(TIF)

S6 Fig Co-localization of CRN78 and PIP2;2 in the plasma membrane. **(A and B)** Co-localization of CRN78 and nucleus marker DAPI or membrane marker pm-RK. **(C)** The localization pattern of GFP-CRN78 in plasmolyzed cells. Samples were plasmolyzed by 800 mM mannitol. Asterisks indicate areas between plasmolyzed cells. Hechtian strands are noticeable in leaves expressing GFP-CRN78 but not in those expressing GFP. Nucleus was labeled by white arrows. **(D)** Interaction between CRN78 and NbPIP2;2 in the BiFC assay. *A*. *tumefaciens* cells harboring cYFP-CRN78 and NbPIP2;2-nYFP were co-infiltrated into *N*. *benthamiana* leaves. YFP fluorescence was observed 48 hours after infiltration. nYFP, cYFP, and NbPIP1;1-nYFP were used as controls. Scale bars: 20 μm. **(E)** CRN78 interacts with NbPIP2;2 at the plasma membrane. Membrane marker pm-RK was co-expressed with cYFP-CRN78 and NbPIP2;2-nYFP, and fluorescence was observed 48 hours after infiltration in no-plasmolysis or plasmolysis cells. Scale bars: 20 μm.
(TIF)

S7 Fig PIP2;2 acts as a H_2_O_2_ transporter. **(A)** Relative transcript levels of *NbPIP2;2* in *NbPIP2;2*-silenced lines. Transcript levels of *NbPIP2;2* were analyzed by qRT-PCR. The actin gene was used as an internal reference. Bars represent standard errors from three independent biological replicates (mean ± SD; n = 3; **, *P* < 0.01 compared with the GFP-silenced lines; Student’s *t*-test). **(B)** The phenotype of *NbPIP2;2*-silenced lines. Photographs were taken at 2 weeks post infiltration. **(C-D)** Changes in the H_2_O_2_-probing fluorescence densities in *NbPIP2;2*-silenced leaves (**C**) or *NbPIP2;2-*overexpressed leaves (**D**) in 0 min or 30 min after H_2_O_2_ treatment. (E-F) Changes in the AR or AUR fluorescence densities in leaves expressing NbPIP2;2 in 30 min after mock treatment.
(TIF)

S8 Fig Apoplast and cytoplast H_2_O_2_ accumulation in leaves upon flg22 treatment. **(A and B)** Changes in the AR or AUR fluorescence densities in leaves expressing NbPIP2;2 in 30 min after flg22 treatment. **(C and D)** H_2_O_2_-probing fluorescence densities in NbPIP2;2-silenced leaves in 30 min after flg22 treatment.
(TIF)

S9 Fig Putative phosphorylation site in NbPIP2;2. Sequence alignment of all eight AtPIPs with NbPIP2;2 was conducted. Phosphorylation sites identified by Mergner *et al* [34] and Rodrigues *et al* [30] are indicated by the red box and the four phosphorylation sites we selected to construct NbPIP2;2^4A^ are indicated with red arrows.
(TIF)

S10 Fig Sequence alignment of NbPIP2;2 with its mutants. Sequence alignment of NbPIP2;2 with its mutants was performed by using the MUSCLE software.
(TIF)

S11 Fig CRN78 associates with AtPIP2;4/5/7/8. **(A-B)** Luciferase complementation assays were performed on *N*. *benthamiana* plants. Chemiluminescence signals were detected at 48 hours post infiltration (**A**). Error bars indicate SD. Proper protein expression is shown on the right (**B**).
(TIF)

S12 Fig Reduced PIP2;2 protein levels in the presence of CRN78. **(A)** PIP1;1-RFP fluorescence was not changed in the presence of CRN78. Confocal microscopy images were taken at 48 hours post infiltration. **(B)** Protein levels from the same sample shown in Fig 6B were analyzed by immunoblotting. Numbers below the blots represent the relative abundance of NbPIP2;2-RFP or NbPIP1;1-RFP. Ponceau staining was used to show equal loading.
(TIF)

S13 Fig CRN78 is conserved in *Phytophthora* and downy mildew. Sequence alignment of five proteins from *P*. *sojae*, *P*. *megakrarya*, *P*. *capsici*, and *P*. *effuse* was conducted. The LXLFLAK motif and conserved kinase domains were labeled.
(TIF)

S1 Data Phospho-peptide detected in GFP- and GFP-CRN78 transgenic *Arabidopsis* plants. (XLSX)

S2 Data Overall counts of PIP2;2-like proteins per species. (XLSX)

S3 Data General information of PIP2;2-like proteins. (XLSX)

S4 Data Primers used in this study. (XLSX)

S5 Data Excel spreadsheet containing, in separate sheets, the underlying numerical data. (XLSX)
