# Supplementary figures and images for: A Phytophthora sojae CRN effector mediates phosphorylation and degradation of plant aquaporin proteins to suppress host immune signaling
Source: PLoS Pathog. 2021 Mar 12;17(3):e1009388. doi: 10.1371/journal.ppat.1009388 (PMC7990189; doi:10.1371/journal.ppat.1009388)

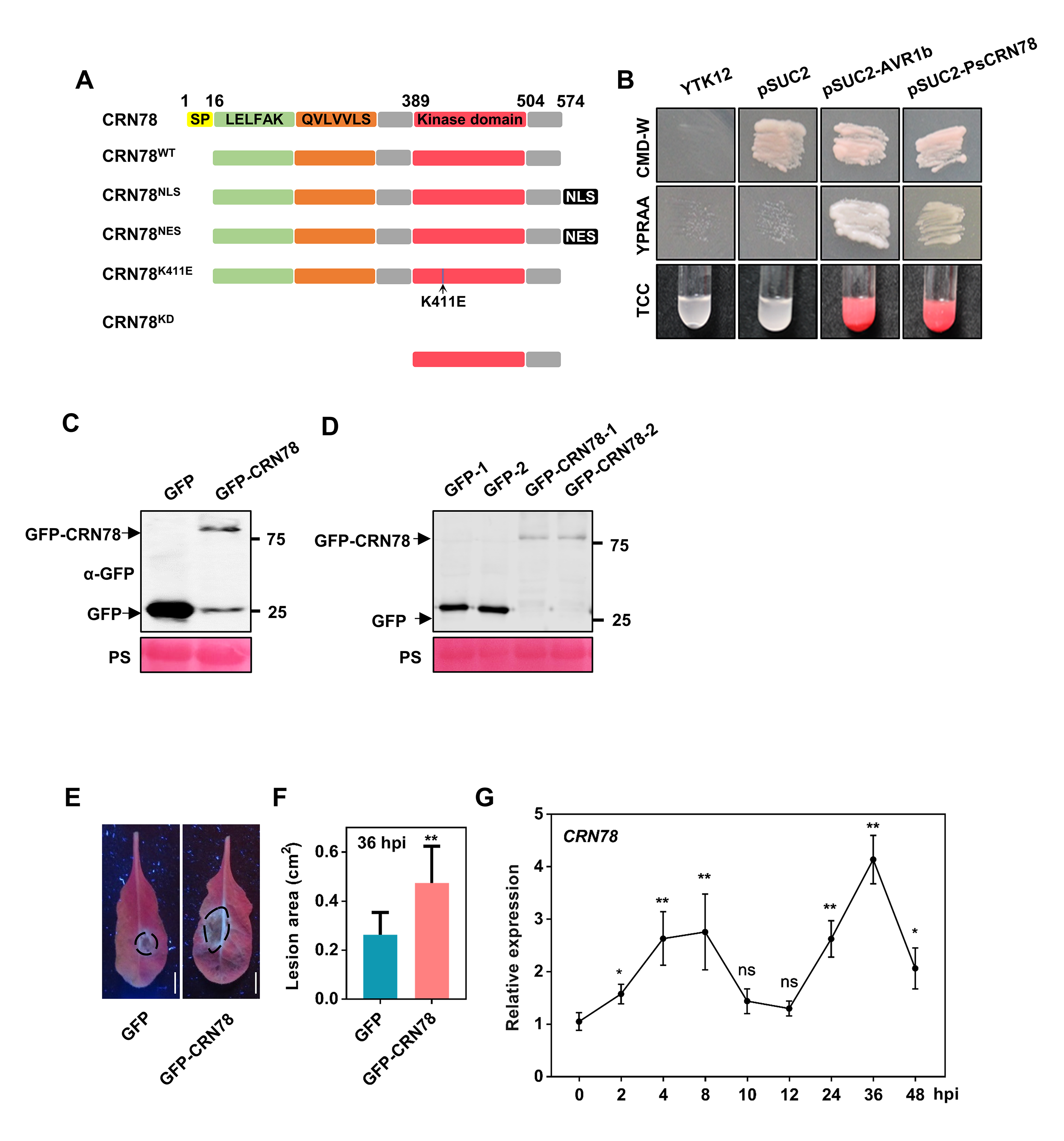

Supplement: S1 Fig — (A) Diagram of motifs in CRN78 and CRN78 mutants constructed in this study. (B) Yeast invertase secretion assay of the predicted CRN78 signal peptide. (C) Immunoblot analysis. The α-GFP antibody was used to detect expression of the indicated constructs. Equal loading of each sample is indicated by Ponceau staining of the Rubisco protein (PS). (D-F) Enhanced P. capsici infection in leaves expressing CRN78. 500 P. capsici zoospores were inoculated onto the leaves expressing GFP or GFP-CRN78. Photographs (E) were taken at 36 hpi under UV light. Lesion areas (F) were calculated from three independent biological replicates with at least five leaves per replicate (mean ± SD; n > 16; **, P < 0.01, Student’s t-test). (G) Relative transcript levels of CRN78 during infection. Hypocotyls of etiolated soybean cv. Williams were challenged with zoospores of P. sojae, and RNA was extracted at indicated times. The transcript levels of CRN78 in P. sojae were normalized to the levels of PsACTIN gene. (mean ± SD; n = 3, *, P < 0.05, **, P < 0.01 compared with the sample of 0 hpi; Student’s t-test). (TIF) [file ppat.1009388.s001.tif]

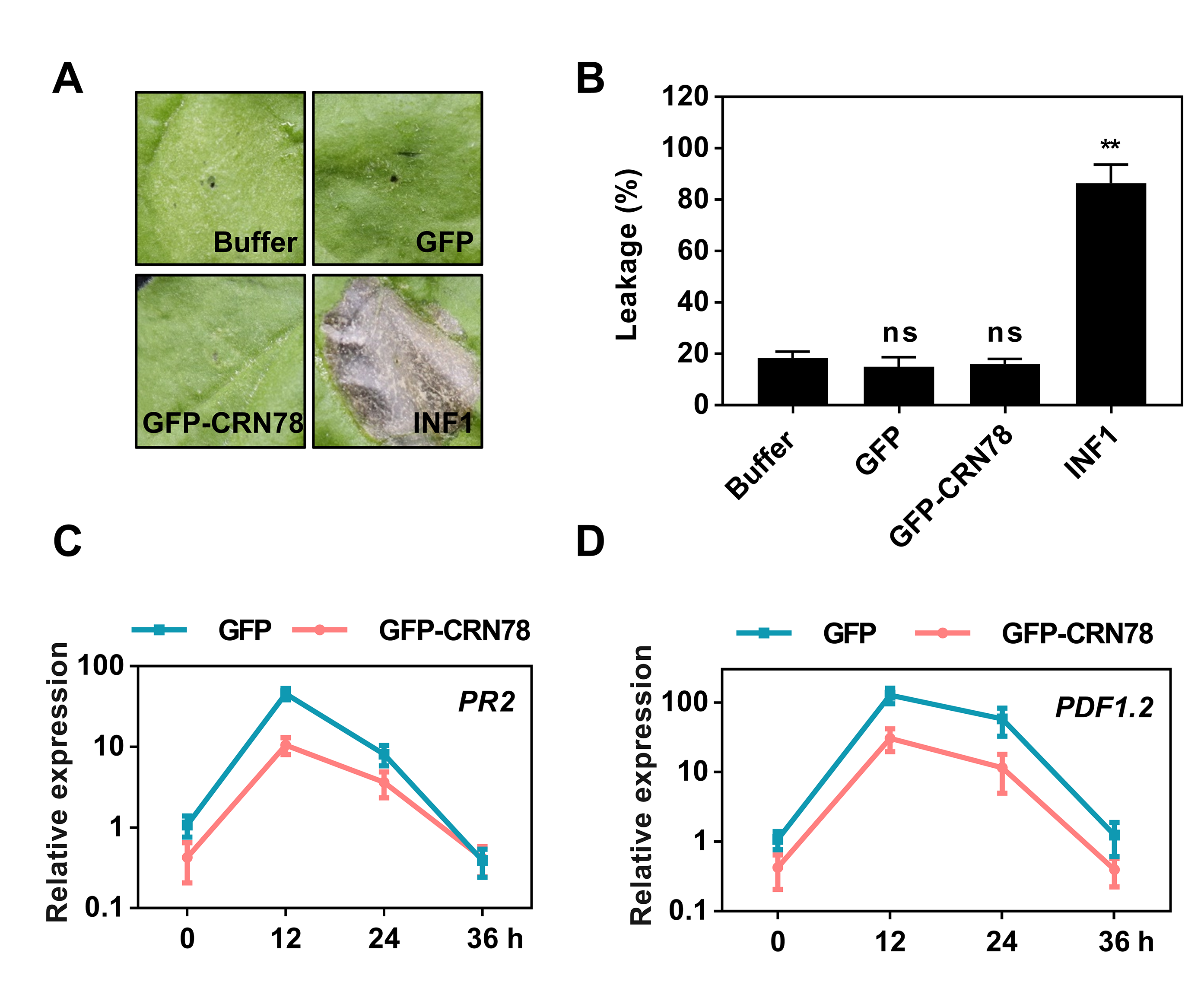

Supplement: S2 Fig — (A) CRN78 did not induce cell death in leaves of N. benthamiana. (B) Ion leakage assay in leaves expressing CRN78. (C and D) Relative transcript levels of NbPR2 and NbPDF1.2. The transcript levels of NbPR2 and NbPDF1.2 in N. benthamiana leaves expressing GFP or CRN78 were analyzed by qRT-PCR with actin as the internal reference (mean ± SD; n = 3). (TIF) [file ppat.1009388.s002.tif]

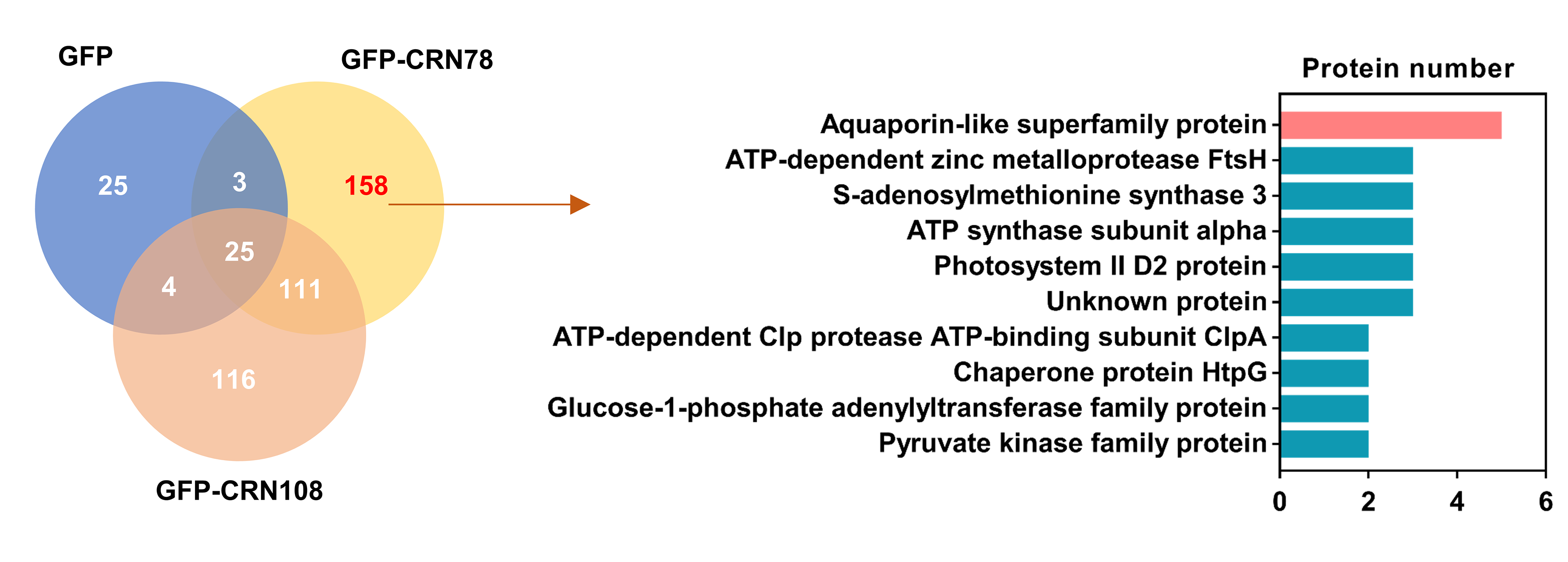

Supplement: S3 Fig — Venn diagram of putative interactor of GFP/GFP-CRN78/GFP-CRN108 is shown on the left. The Protein frequencies of top 10 super families are shown on the right. (TIF) [file ppat.1009388.s003.tif]

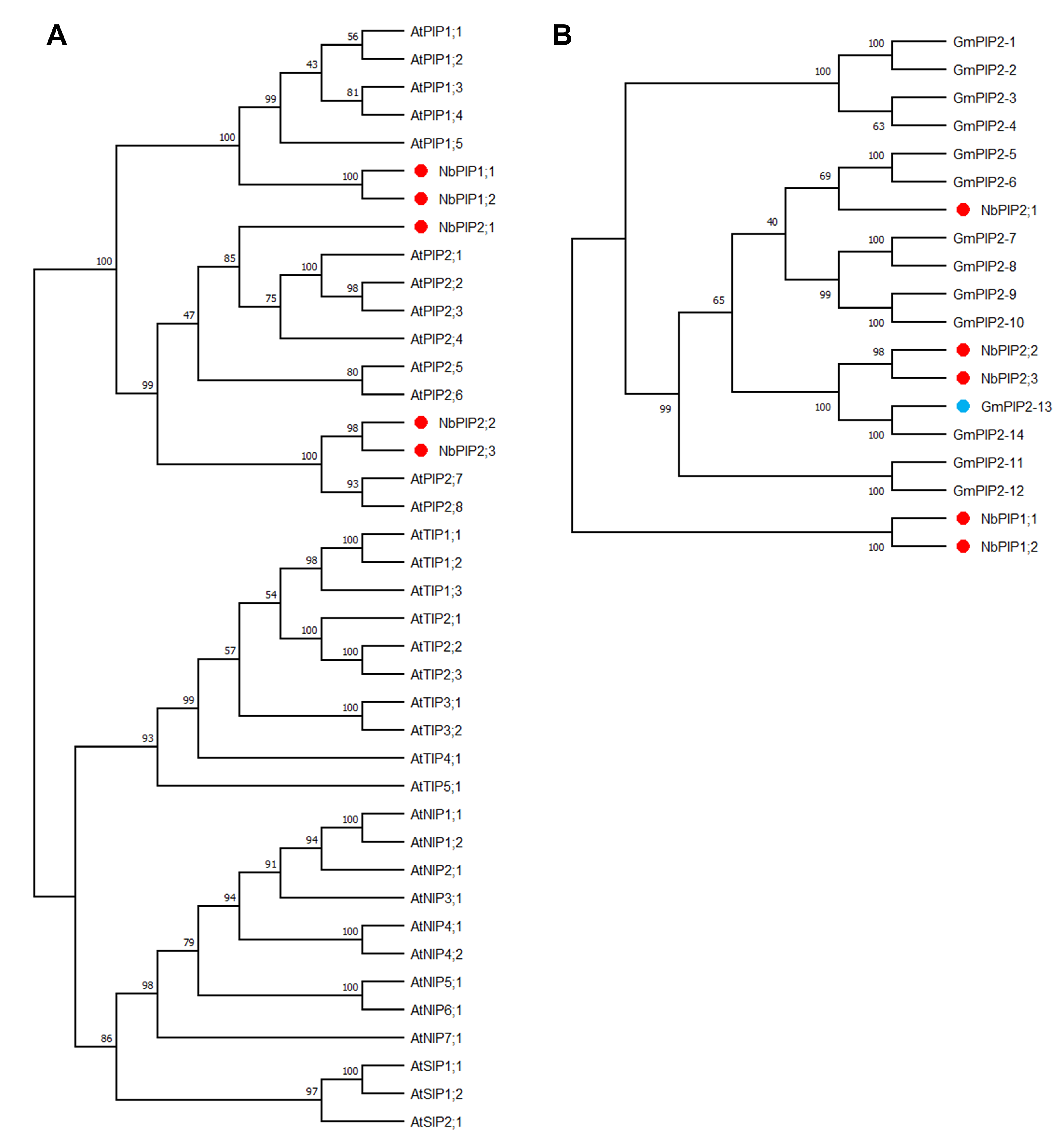

Supplement: S4 Fig — (A) The phylogenetic tree of five NbPIPs identified in mass spectrum data with all aquaporins reported in Arabidopsis. The five NbPIPs is indicated in red. (B) The phylogenetic tree of five NbPIPs with all PIP2 proteins from soybean. The five NbPIPs are indicated in red and GmPIP2-13 is indicated in blue. (TIF) [file ppat.1009388.s004.tif]

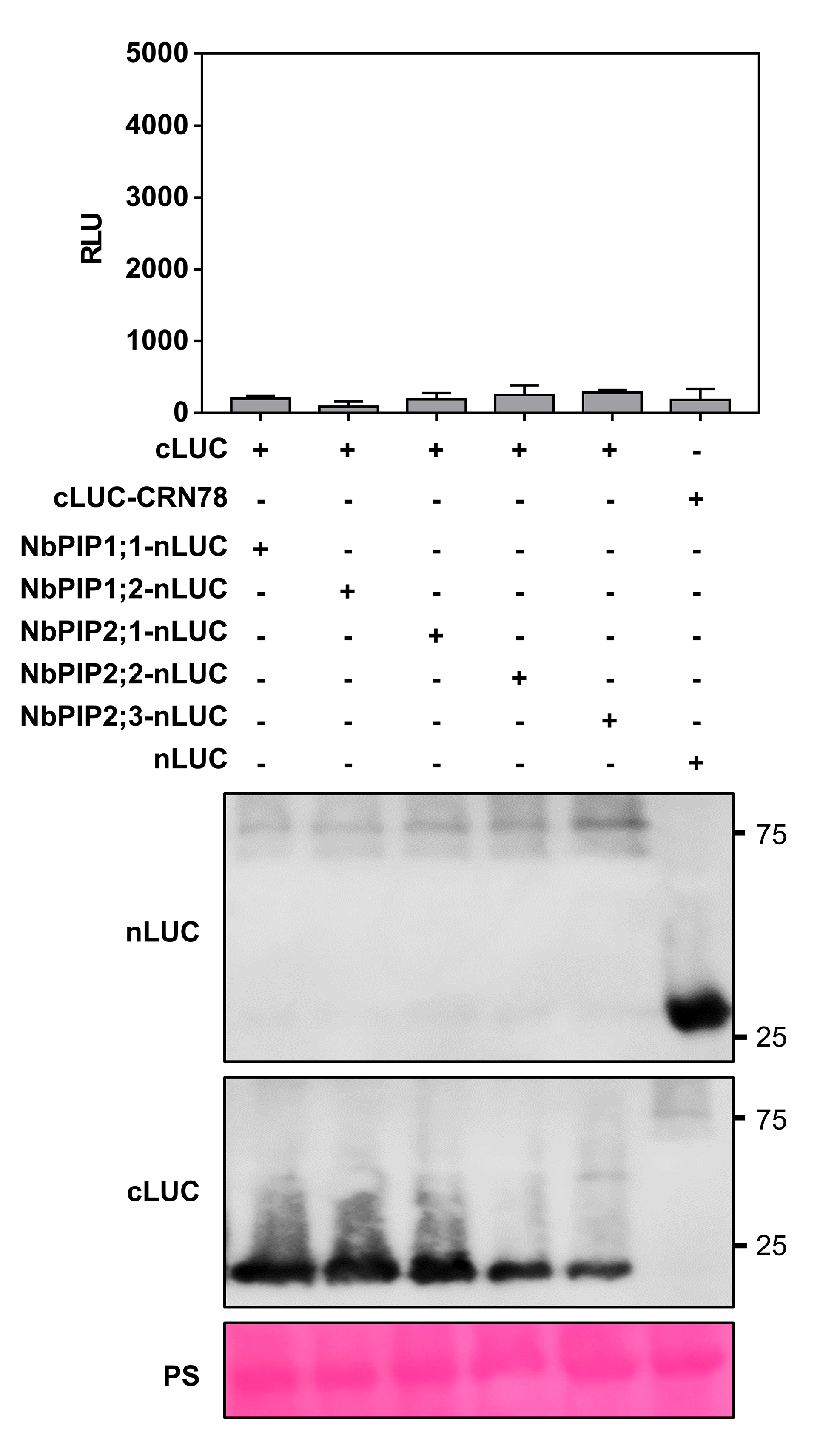

Supplement: S5 Fig — Luciferase complementation assay was performed on N. benthamiana plants by Agrobacterium-mediated transient expression of the indicated constructs. Relative luminescence units (RLU) of each combination are shown at the top (mean ± SD; n = 3). Proper protein expression is shown at the bottom. (TIF) [file ppat.1009388.s005.tif]

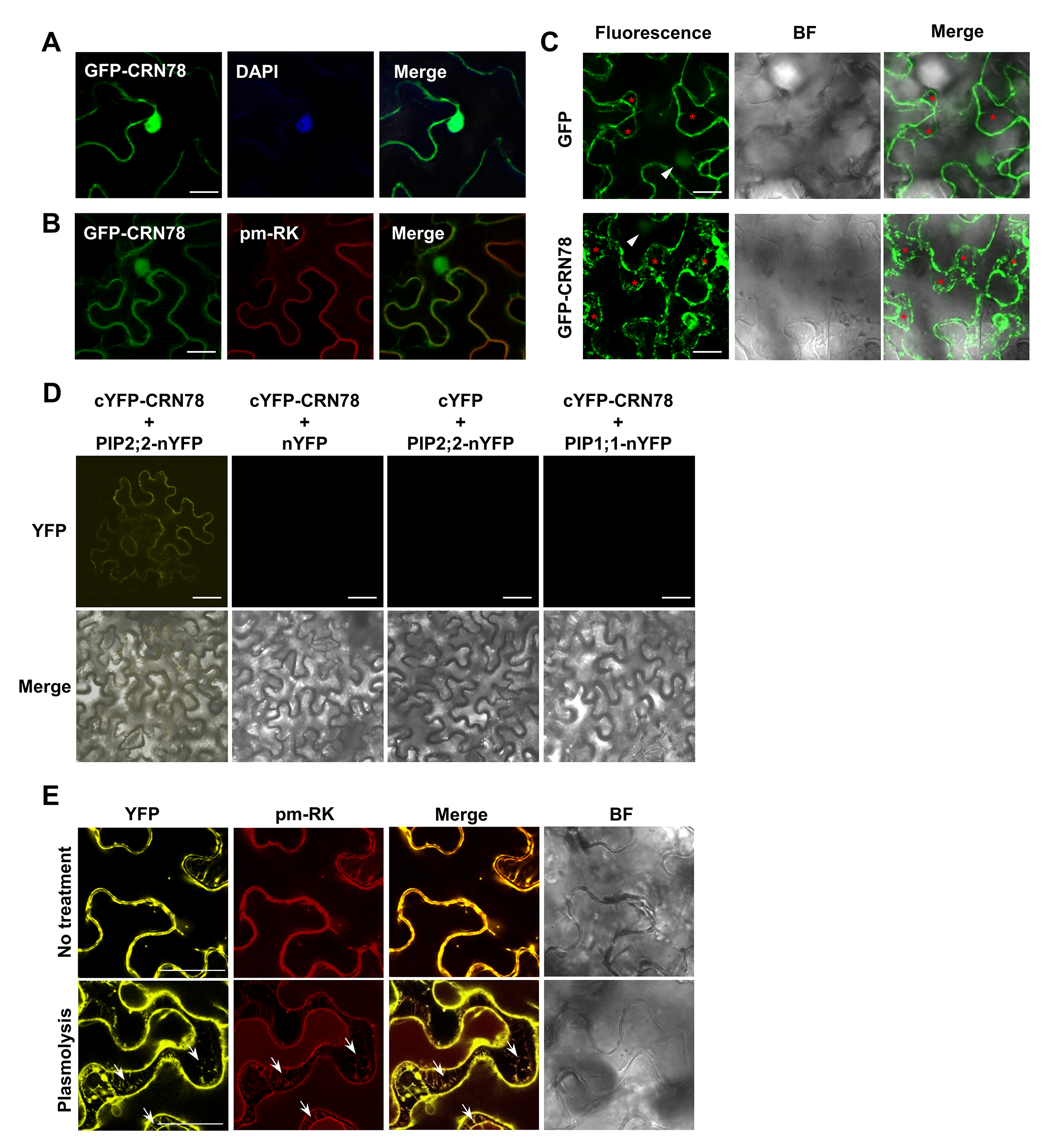

Supplement: S6 Fig — (A and B) Co-localization of CRN78 and nucleus marker DAPI or membrane marker pm-RK. (C) The localization pattern of GFP-CRN78 in plasmolyzed cells. Samples were plasmolyzed by 800 mM mannitol. Asterisks indicate areas between plasmolyzed cells. Hechtian strands are noticeable in leaves expressing GFP-CRN78 but not in those expressing GFP. Nucleus was labeled by white arrows. (D) Interaction between CRN78 and NbPIP2;2 in the BiFC assay. A. tumefaciens cells harboring cYFP-CRN78 and NbPIP2;2-nYFP were co-infiltrated into N. benthamiana leaves. YFP fluorescence was observed 48 hours after infiltration. nYFP, cYFP, and NbPIP1;1-nYFP were used as controls. Scale bars: 20 μm. (E) CRN78 interacts with NbPIP2;2 at the plasma membrane. Membrane marker pm-RK was co-expressed with cYFP-CRN78 and NbPIP2;2-nYFP, and fluorescence was observed 48 hours after infiltration in no-plasmolysis or plasmolysis cells. Scale bars: 20 μm. (TIF) [file ppat.1009388.s006.tif]

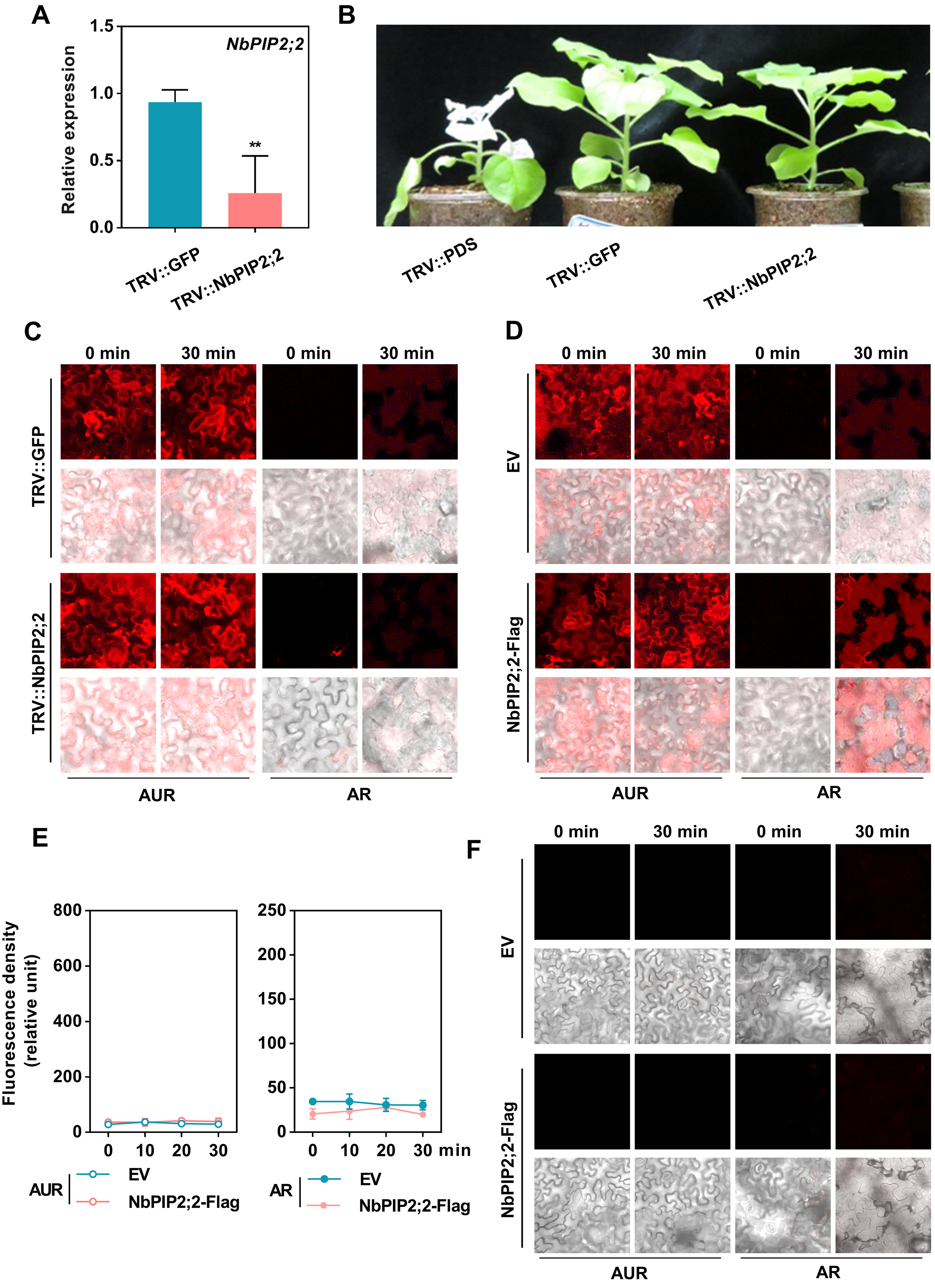

Supplement: S7 Fig — (A) Relative transcript levels of NbPIP2;2 in NbPIP2;2-silenced lines. Transcript levels of NbPIP2;2 were analyzed by qRT-PCR. The actin gene was used as an internal reference. Bars represent standard errors from three independent biological replicates (mean ± SD; n = 3; **, P < 0.01 compared with the GFP-silenced lines; Student’s t-test). (B) The phenotype of NbPIP2;2-silenced lines. Photographs were taken at 2 weeks post infiltration. (C-D) Changes in the H2O2-probing fluorescence densities in NbPIP2;2-silenced leaves (C) or NbPIP2;2-overexpressed leaves (D) in 0 min or 30 min after H2O2 treatment. (E-F) Changes in the AR or AUR fluorescence densities in leaves expressing NbPIP2;2 in 30 min after mock treatment. (TIF) [file ppat.1009388.s007.tif]

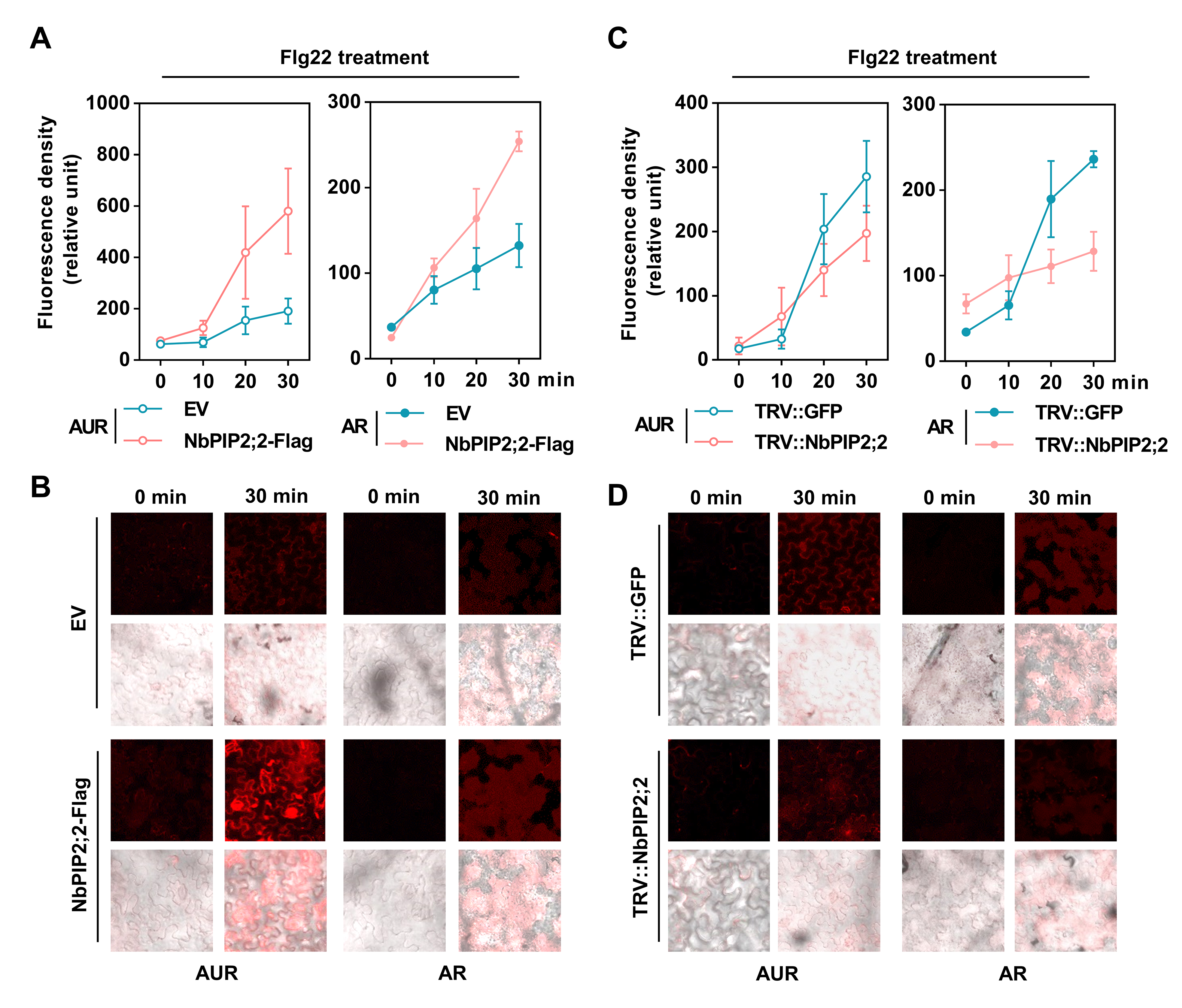

Supplement: S8 Fig — (A and B) Changes in the AR or AUR fluorescence densities in leaves expressing NbPIP2;2 in 30 min after flg22 treatment. (C and D) H2O2-probing fluorescence densities in NbPIP2;2-silenced leaves in 30 min after flg22 treatment. (TIF) [file ppat.1009388.s008.tif]

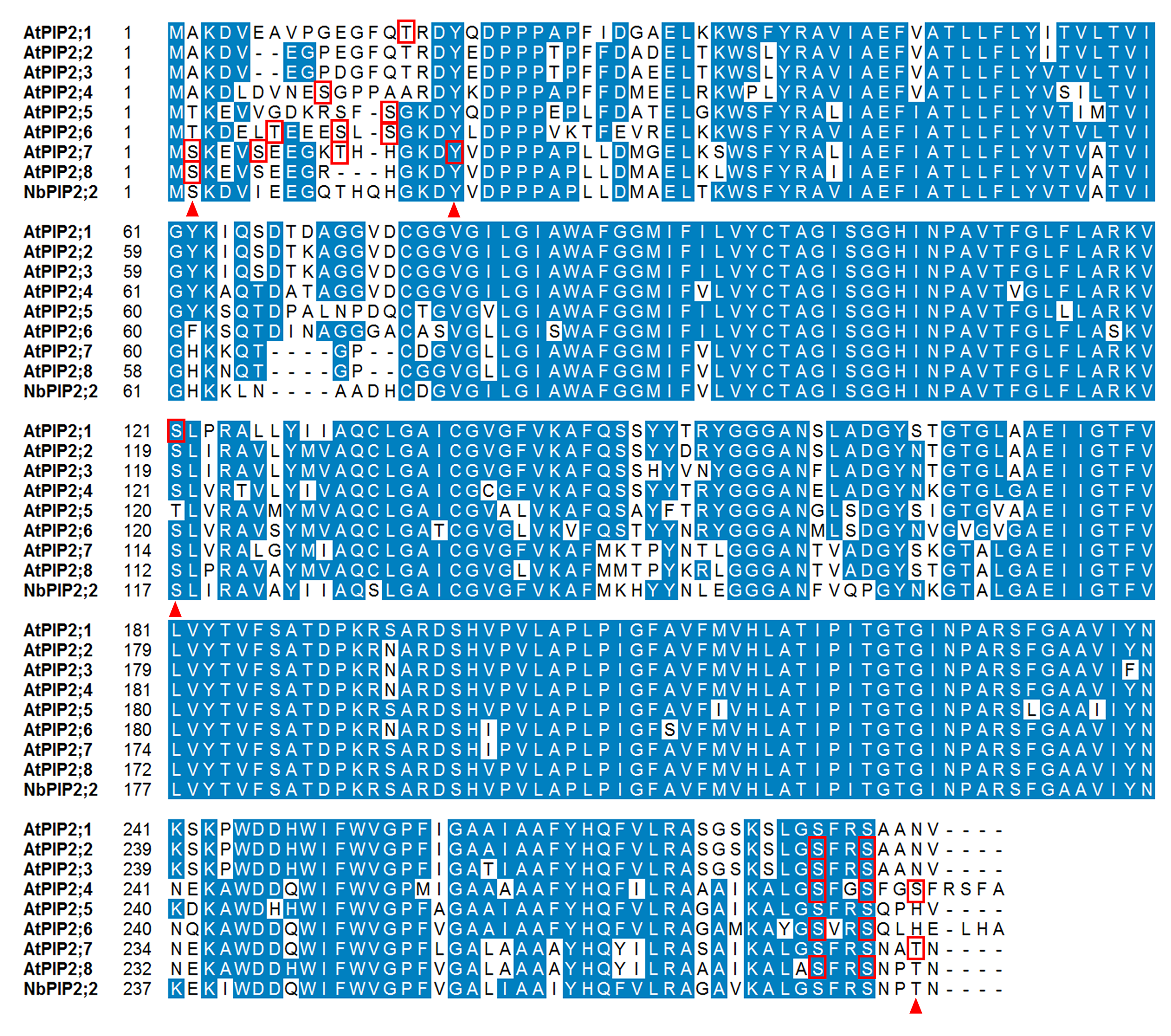

Supplement: S9 Fig — Sequence alignment of all eight AtPIPs with NbPIP2;2 was conducted. Phosphorylation sites identified by Mergner et al [34] and Rodrigues et al [30] are indicated by the red box and the four phosphorylation sites we selected to construct NbPIP2;24A are indicated with red arrows. (TIF) [file ppat.1009388.s009.tif]

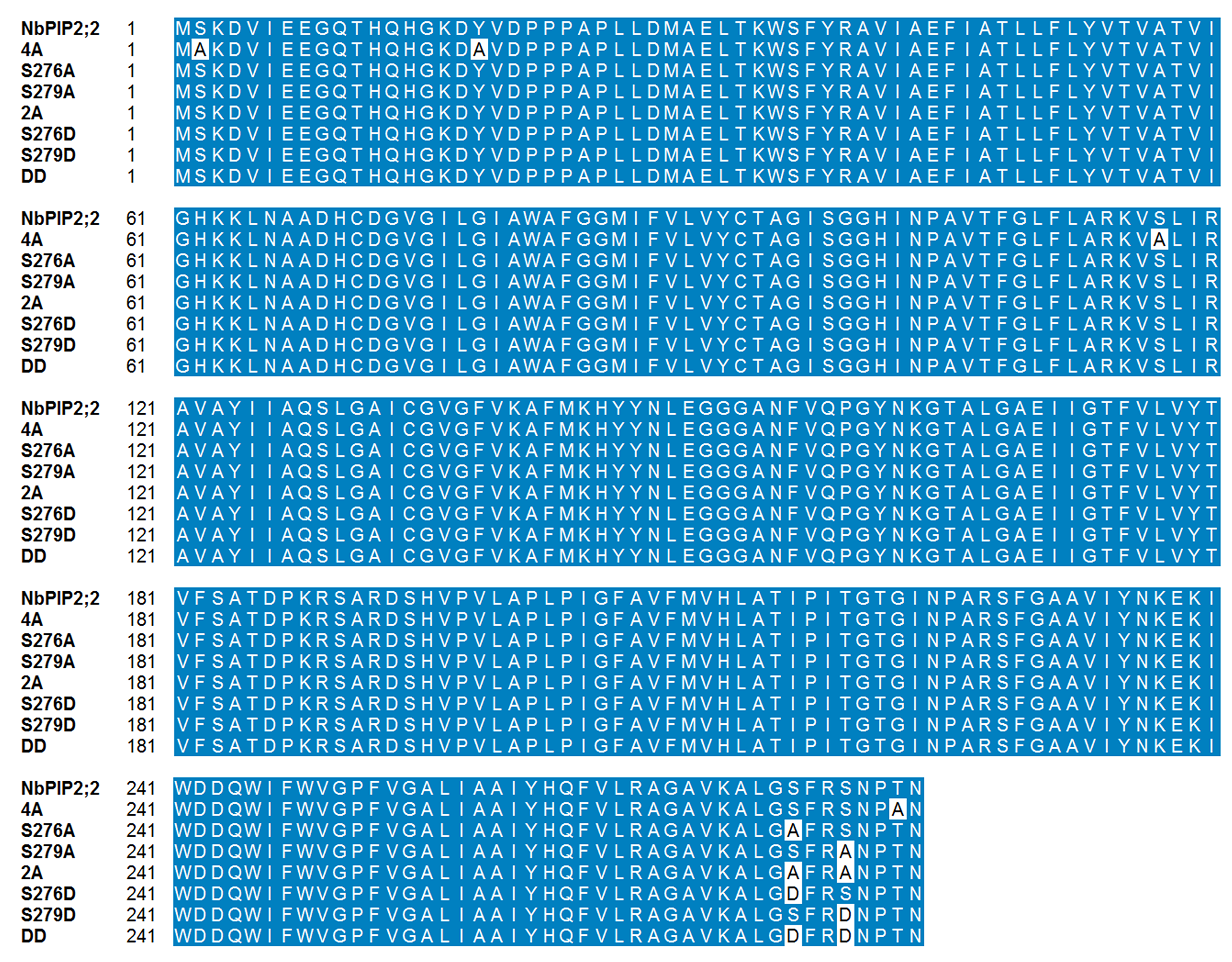

Supplement: S10 Fig — Sequence alignment of NbPIP2;2 with its mutants was performed by using the MUSCLE software. (TIF) [file ppat.1009388.s010.tif]

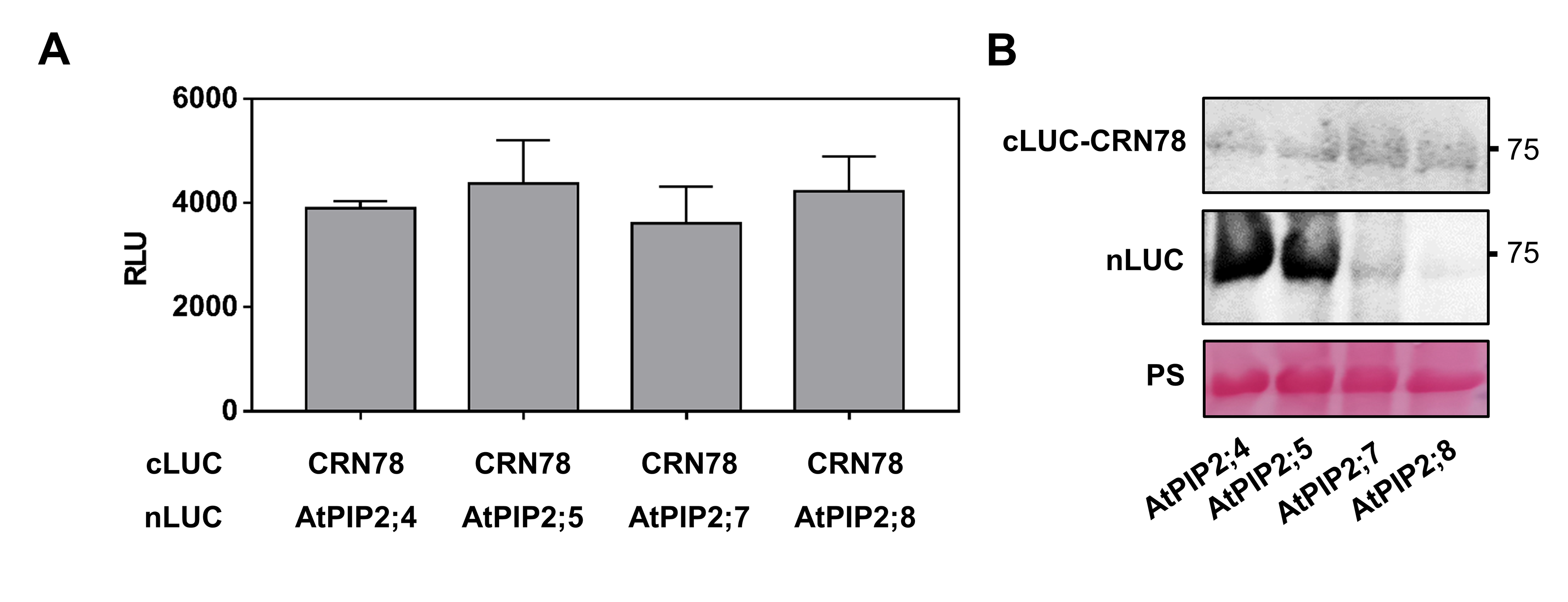

Supplement: S11 Fig — (A-B) Luciferase complementation assays were performed on N. benthamiana plants. Chemiluminescence signals were detected at 48 hours post infiltration (A). Error bars indicate SD. Proper protein expression is shown on the right (B). (TIF) [file ppat.1009388.s011.tif]

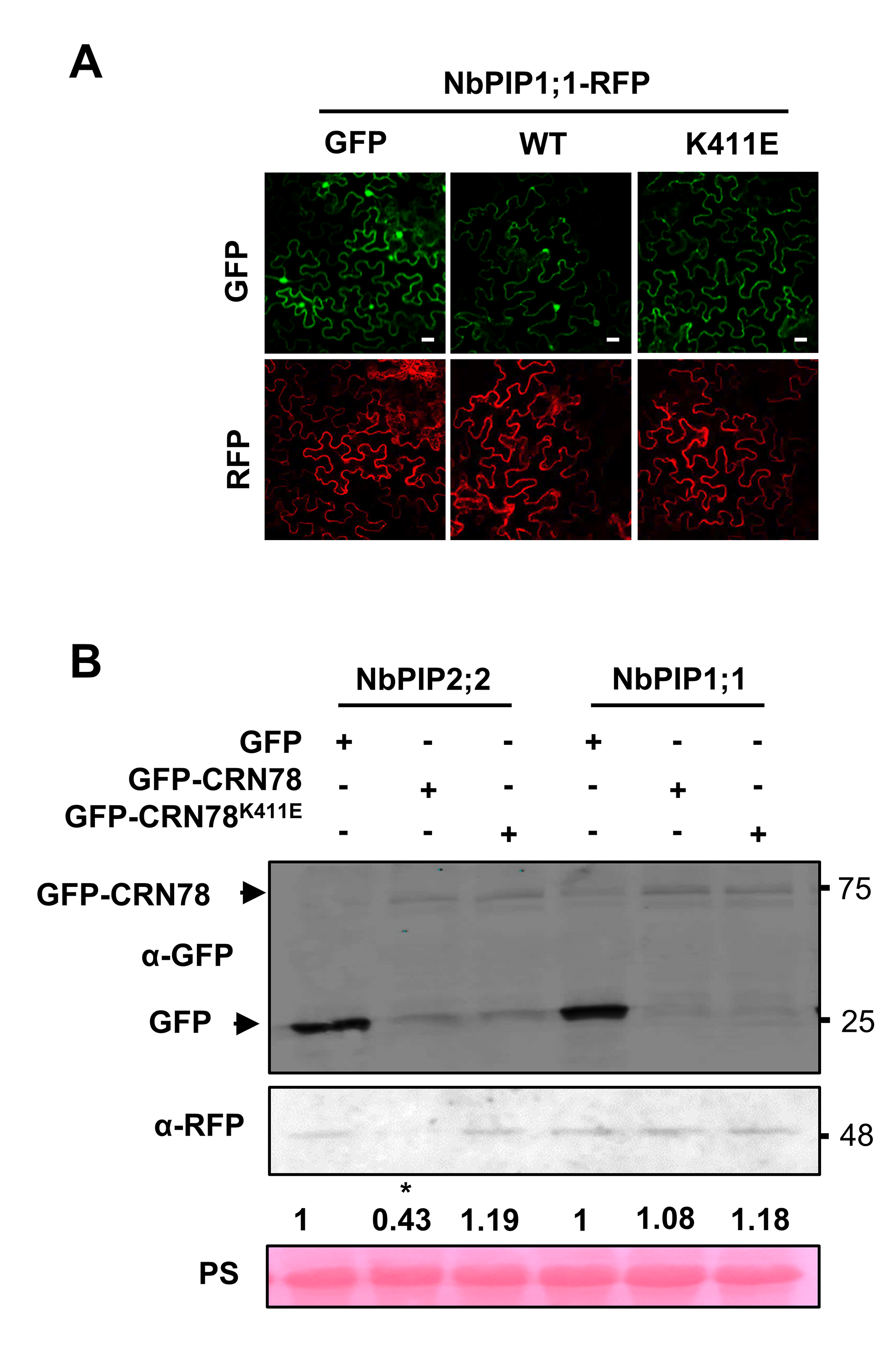

Supplement: S12 Fig — (A) PIP1;1-RFP fluorescence was not changed in the presence of CRN78. Confocal microscopy images were taken at 48 hours post infiltration. (B) Protein levels from the same sample shown in Fig 6B were analyzed by immunoblotting. Numbers below the blots represent the relative abundance of NbPIP2;2-RFP or NbPIP1;1-RFP. Ponceau staining was used to show equal loading. (TIF) [file ppat.1009388.s012.tif]

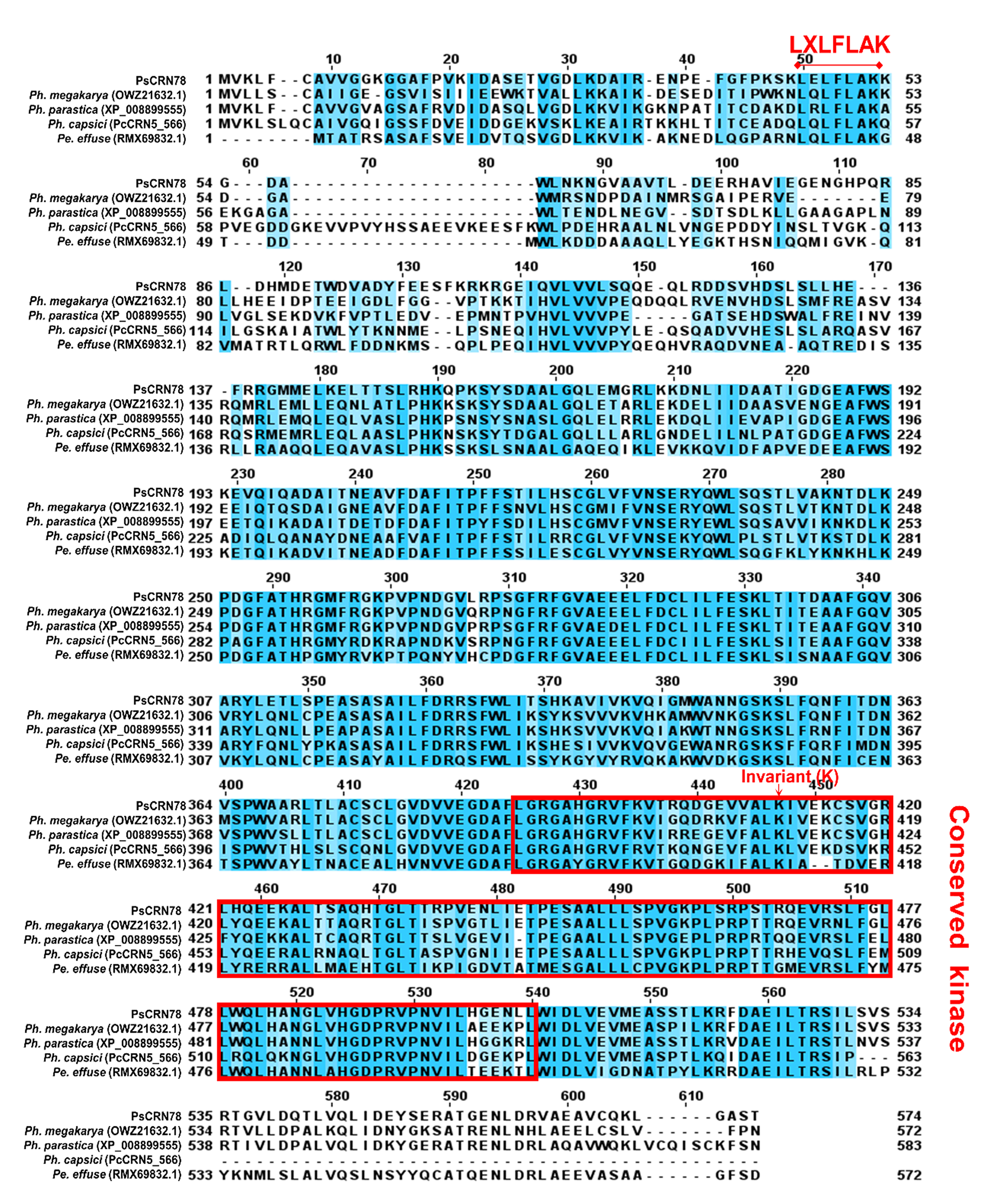

Supplement: S13 Fig — Sequence alignment of five proteins from P. sojae, P. megakrarya, P. capsici, and P. effuse was conducted. The LXLFLAK motif and conserved kinase domains were labeled. (TIF) [file ppat.1009388.s013.tif]
